# A meta-analysis of healthy lifestyle interventions addressing quality of life of cancer survivors in the post treatment phase

**DOI:** 10.1007/s11764-023-01514-x

**Published:** 2024-01-11

**Authors:** Morgan Leske, Christina Galanis, Bogda Koczwara, Lisa Beatty

**Affiliations:** 1 College of Education, Psychology, and Social Work, Flinders University, Adelaide, SA Australia; 2 College of Medicine and Public Health, Flinders University, Adelaide, SA Australia; 3 Department of Medical Oncology, Southern Adelaide Local Health Network, Adelaide, SA Australia

**Keywords:** Cancer survivors, Lifestyle intervention, Quality of life, Complex interventions

## Abstract

**Purpose:**

This study’s primary aim was to investigate whether including a mental health component to healthy lifestyle interventions are associated with greater effects on quality of life (QoL) for post-treatment cancer survivors than addressing physical activity and/or nutrition alone.

**Methods:**

PsycINFO, Scopus, Medline, CINAHL, and Google Scholar were searched to identify randomised control trials of healthy lifestyle interventions for post-treatment cancer survivors, with a usual care or waitlist control, and measured QoL. Meta-analyses quantified the effects of interventions vs controls at post-treatment on total QoL, physical, emotional, and social well-being. Subgroup analyses compared interventions with vs without a mental health component, modes of delivery, and duration. The quality of the included studies was assessed using the Cochrane Risk of Bias 2.

**Results:**

Eighty-eight papers evaluating 110 interventions were included: 66 effect sizes were extracted for meta-analysis, and 22 papers were narratively synthesised. The pooled effect size demonstrated a small, significant effect of healthy lifestyle interventions in comparison to control for all QoL outcomes (total *g* = 0.32, *p* >.001; physical *g* = 0.19, *p* = 0.05; emotional *g* = 0.20, *p* >.001; social *g* = 0.18, *p* = 0.01). There was no significant difference between interventions with vs without a mental health component. Face-to-face delivered interventions were associated with greater total QoL and physical well-being compared to other modalities. Interventions delivered ≤12 weeks were associated with greater physical well-being than those delivered ≥13 weeks. Overall, studies had substantial levels of heterogeneity and 55.9% demonstrated high risk of bias.

**Conclusions:**

Participating in a healthy lifestyle intervention following cancer treatment improves QoL. Few trials addressed mental health or evaluated online or telephone modalities; future research should develop and evaluate interventions that utilise these features.

**Implications for Cancer Survivors:**

Brief healthy lifestyle interventions can be recommended for cancer survivors, particularly those interested in improving physical well-being.

**Supplementary Information:**

The online version contains supplementary material available at 10.1007/s11764-023-01514-x.

## Introduction

Advances in earlier detection and diagnosis, improved treatment options, and better supportive care are contributing to the growing cancer survivor population [1]. However, the physical (e.g. fatigue, pain, nausea, and changes in appearance) and psychosocial (e.g. psychological distress, challenges in relationships, financial stress, and changes in cognitive and sexual functioning) side effects of a cancer diagnosis and its associated treatments can significantly impact an individual’s quality of life (QoL) long after they have completed treatment [2–4]. QoL for cancer survivors is a subjective multi-dimensional concept that encompasses and measures various aspects of a person’s physical, emotional, social, and spiritual well-being, and functional status. QoL refers to how a person perceives their life in the context of their health and personal values, and how well they can function and participate in activities that are important to them [5–7].

Healthy lifestyle interventions addressing physical activity, nutrition, and/or weight management have been posited as one strategy to improve QoL and support cancer survivors following the completion of treatment. Such interventions have demonstrated efficacy in (a) reducing treatment-related side effects, cancer recurrence and mortality [8], and (b) improving emotional well-being [9]. Several meta-analyses have evaluated the efficacy of healthy lifestyle interventions in enhancing QoL in cancer survivors, but their results have been inconsistent. Small to moderate positive effects on QoL have been demonstrated across meta-analyses involving physical activity interventions involving all cancer types [7] and breast cancer survivors [9–11]. Similarly, healthy lifestyle education programs have demonstrated a moderate positive effect on lung cancer survivors QoL [12]. In contrast, meta-analyses which have investigated healthy lifestyle interventions for gynaecological cancers [13] or have only involved nutritional therapy [14] have not demonstrated significant differences to usual care control groups. Two meta-analyses investigating telehealth interventions [15, 16], such as those delivered via telephone, or videoconferencing and online platforms, have produced contrasting findings. Larson and colleagues [15] conducted a meta-analysis involving eleven studies and initially obtained a large positive effect; however, the magnitude of the effect was decreased to non-significant when two large studies contributing to heterogeneity were removed. In comparison, the second, and larger, meta-analysis by Li and colleagues [16] involving 28 studies found a small positive effect for telehealth interventions on cancer survivors’ QoL.

Although these meta-analyses support the implementation of healthy lifestyle interventions following cancer treatment, they have primarily focused on interventions which target physical health behaviours, such as physical activity and diet quality. However, a qualitative study conducted by Grant and colleagues [17] with cancer survivors, oncology healthcare professionals, and representatives from cancer support organisations identified that a healthy lifestyle after cancer treatment includes both physical health and mental health. The participants of this study recommended that a mental health component be included in healthy lifestyle interventions. Addressing mental health within healthy lifestyle interventions is also promoted by research investigating barriers to physical activity and healthy eating, which have identified stress as a prevalent barrier to engaging in these health behaviours [18, 19]. 

Thus, interventions targeting a healthy lifestyle after cancer treatment should go beyond physical activity and nutrition and address mental health as well. To date, meta-analyses have not examined whether interventions that include a mental health component increase the impact of healthy lifestyle interventions on cancer survivors’ QoL. The current meta-analysis aims to update the previous evidence for the efficacy of healthy lifestyle interventions on QoL post-intervention and to investigate whether interventions which include a mental health component in their intervention protocol are associated with greater effects on QoL in comparison to interventions which only address physical activity or nutrition. The secondary aim of this meta-analysis is to investigate whether other aspects of the intervention, such as mode of delivery (individual, group, telephone, online, or print) or duration (shorter vs longer), affect the association between the interventions and QoL.

## Method

This meta-analysis followed the Preferred Reporting Items for Systematic Reviews and Meta-Analyses (PRISMA) statement [20] and was prospectively registered on PROSPERO (CRD42021273722).

### Study selection

To identify relevant studies, a review of electronic databases relevant to psychology and health, including PsycINFO, Scopus, Medline, and CINAHL, was conducted. In addition, the first 200 references identified in Google scholar were included in the review. The search strategy was based on the PICO approach, as follows: *population*—terms related to (1) cancer, and (2) survivor; *intervention*—terms related to (1) healthy lifestyle, (2) physical activity, (3) nutrition, and (4) weight control; *outcome*—terms related to QoL (see Multimedia A for details). The final database search was conducted on the 9th of June 2022.

Articles were included in the analysis if they meet the following criteria: (1) involved adult cancer survivors (i.e. ≥18 years and have completed active treatment); (2) offered an intervention targeting health behaviour change (i.e. physical activity, sedentary time, or diet, or weight management); (3) reported an outcome measure for total QoL, and/or Physical, Emotional, or Social Well-being on a reliable and valid measure of QoL (e.g. European Organization for the Research and Treatment of Cancer Quality of Life Questionnaire (EORTC QLQ-C30; [6]), Functional Assessment of Cancer Therapy-General (FACT-G; [5]), or 36- or 12-Item Short Form Health Survey (SF-36; [21], SF-12; [22]); (4) involved a randomised control trial or pilot randomised control trial using a waitlist or usual care control (i.e. access to publicly available materials); (5) written in English and published in a peer-reviewed journal. Included articles investigated interventions utilising any mode of delivery. Articles were excluded if they involved a population other than adult cancer survivors, did not offer an intervention targeting health behaviour change, offered an intervention which only targeted mental health, did not measure QoL, or utilised any of the following designs: crossover design, single group pre-post, qualitative, cross-sectional design, protocol paper, systematic review, or meta-analysis. Articles were also excluded if they were grey literature (e.g. dissertations or conference papers).

Authors ML and CG conducted preliminary screening of titles and abstracts. Abstracts meeting inclusion criteria were subject to full-text evaluation. Disagreement between the two reviewers were resolved through discussion. If consensus was not achieved, a third author (LB) was consulted.

### Data extraction

Data extracted from articles that met inclusion criteria included study characteristics (e.g. author, year of publication, country intervention was delivered), participant characteristics (e.g. gender, age, cancer type, and time since diagnosis), intervention characteristics (i.e. duration, mode of delivery, and behaviours targeted), and outcome measures. Interventions were categorised as addressing physical activity if they targeted bodily movement and included increasing exercise (i.e. planned, structured, and repetitive movements to increase physical fitness), leisure time activity, and reducing sedentary time. Interventions were categorised as addressing nutrition if they targeted the increase and/or decrease of certain foods or nutrients. Interventions were categorised as including a mental health component if they provided a manualised psychological treatment, psycho-education material on mental health and well-being, or counselling with the intention of addressing emotional distress. To calculate effect sizes between the intervention and control groups, the post-intervention sample size, means, and standard deviations for total QoL were extracted. As several QoL measures do not quantify a total score, the means and standard deviations of subscales relevant for physical, emotional, and social well-being in both the intervention and control groups were also extracted. These subscales were selected as they were present in all valid QoL scales. For inter-rater reliability, two authors (ML and CG) undertook data extraction on a subset of articles (*n* = 58).

### Quality assessment

The risk of bias of each study was evaluated by one author (ML) using the Cochrane Risk of Bias tool 2.0 (RoB 2; [23]). This tool evaluates the risk of bias in five domains: (1) the randomisation process, (2) deviations from intended interventions, (3) missing outcome data, (4) measurement of outcome, and (5) selection of the reported result. As the current meta-analysis was summarising self-reported QoL, domain 4: measurement of outcome, was not considered in the evaluation of risk. Using this tool, the articles were evaluated and judged on the domains as being either low risk of bias, some concerns, or high risk of bias. For overall bias, articles were considered to have low risk of bias if they were rated as low risk of bias on each of the domains and high risk of bias if they were rated as having high risk of bias on at least one of the domains or as having some concerns on at least two of the domains.

### Data analysis

The Comprehensive Meta-Analysis computer package [24] was used for all analyses. Standardised mean differences (Hedge’s *g*) between the intervention and control groups with 95% confidence intervals were calculated for the total QoL and each of the QoL subscales. Effect sizes were pooled using a random effects model to derive the overall effect size of healthy lifestyle interventions on QoL for cancer survivors. Following this, three pre-specified subgroup analyses were conducted to investigate whether the efficacy of healthy lifestyle interventions on QoL was influenced by selected intervention components. The first subgroup analysis interventions were categorised based on the inclusion of a mental health component. The second sub-group analysis separated interventions based on their dominant mode of delivery, such as individual face-to-face, groups, telehealth, digital health, or print. As there were interventions where one delivery was not dominant, a multiple category was included. The final pre-specified sub-group analysis investigated interventions which had a shorter duration (i.e. 12 weeks or less) or a longer duration (i.e. 13 weeks or more). Narrative synthesis was used to summarise findings in studies which could not be included in the meta-analysis. The narrative synthesis focused on the efficacy of the healthy lifestyle intervention in comparison to the usual care control and the potential impact the intervention characteristics of the inclusion of a mental health component, the mode of intervention delivery, and intervention duration.

#### Heterogeneity and publication bias

The heterogeneity of the data was assessed using *Q* (presence of heterogeneity) and *I*^2^ (proportion of total variation between studies that results from heterogeneity) statistics [25]. The *I*^2^ scale ranges from 0% (no heterogeneity) to 100% (high heterogeneity). Cochrane’s guide to interpretation of the *I*^2^ statistic specifies that 0–40% = heterogeneity that might not be important, 30–60% = moderate heterogeneity, 50–90% = substantial heterogeneity, and 75–100% = considerable heterogeneity. To interpret the *I*^2^ statistic, the number of studies included magnitude and direction of the effect, and *Q* statistic was taken into consideration. Sources of heterogeneity were explored by conducting post hoc sub-group analyses [26], by dividing studies into two or more subgroups and calculating the *Q* and *I*^2^ statistics for each subgroup. Three subgroups were explored: (1) multi-component (i.e. targeting more than one health behaviour) vs single component (i.e. targeting a single health behaviour); (2) measure of QoL; (3) QoL measured as the primary vs secondary outcome. For the second subgroup analysis, the measures of QoL were grouped under their measurement system, rather than individual measures, to ensure relatively equal groups. For example, those who included the FACT-Breast, FACT-Colorectal, and FACT-General were grouped under FACT and the SF12 and SF-36 were grouped under SF.

Publication bias was evaluated by Egger’s regression intercept, which examines the correlation between effect sizes and standard errors of effect sizes. If there is a significant association between study effect size and study precision, this indicates the possibility of publication bias. Each QoL outcome was considered separately.

## Results

### Study selection

**Fig. 1:**
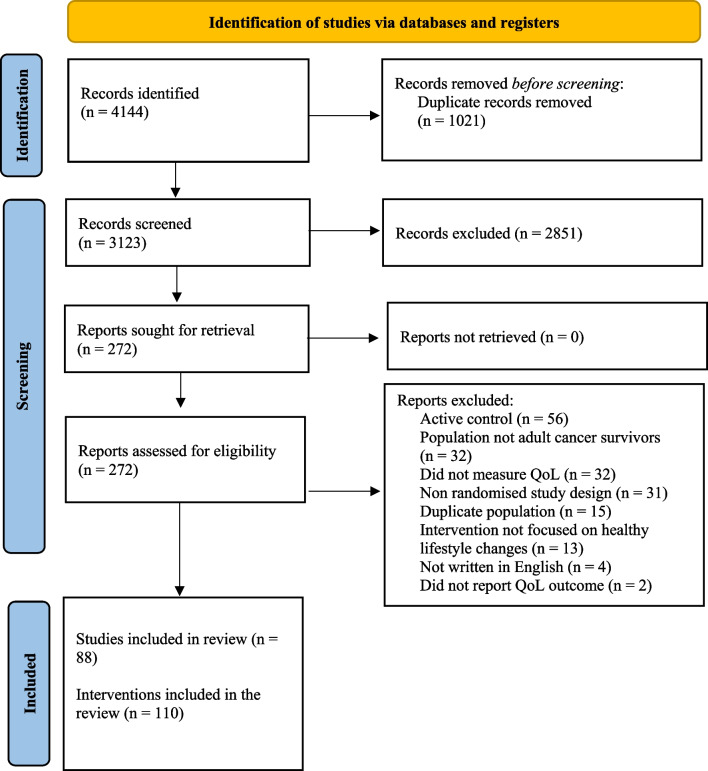
PRISMA flow diagram of included studies

Figure 1 presents the PRISMA flow diagram of the study selection process. Following screening, 88 articles involving 110 interventions met inclusion criteria for the systematic review, and 66 articles met criteria for meta-analysis. Articles were most commonly excluded due to the use of an active control (e.g. workbook or telephone calls). The predominant reason for excluding articles from the meta-analysis was reporting change over time instead of post treatment means and standard deviations. The agreement rate between reviewers was 91.5% for title and abstract screening, 77.4% for full text review, and 66% for data extraction. Exacting different total scores for QoL when multiple scales were reported (e.g. SF-36 and FACT-G) accounted for 73% of the differences in the data extraction. In all instances of disagreement, consensus was reached through discussion.

### Study characteristics

Multimedia B summarises the 88 included studies. The total number of participants included in this review was 9556, with sample sizes ranging from 14 to 641 and a median of 71. There was an over-representation of females in included studies with 51 interventions offered only to breast cancer survivors. The average age of included participants was 57.93 (*SD* = 11.32) years. Forty-eight studies reported time since diagnosis, of which the median was 23.53 months (range = 6.40–87.6 months). The majority of included studies were conducted in the USA (*n* = 27), Canada (*n* = 11), Australia (*n* = 9), Spain (*n* = 6), Netherlands (*n* = 6), and the UK (*n* = 5). In terms of study design, 30.7% studies measured QoL as their primary outcome. The most common QoL measures were the FACT (*n* = 33), EORTC QLQC30 (*n* = 25), and the SF questionnaire (*n* = 23).

### Intervention characteristics

#### Mode of delivery

A diverse range of delivery modalities were investigated in the included interventions. Most utilised *face-to-face delivery* (*n* = 84), of which approximately half (*n* = 43) were provided individually [27–52] while the remainder were delivered via groups [43, 53–83]. Twenty-five (22.7%) of these face-to-face interventions were supported by additional modalities, such as printed or emailed materials [55–57, 84–86], telephone [41, 66, 87, 88], videos [89, 90], or a combination of these [71, 73, 91].

Sixteen studies utilised a *digital health modality* (such as an online platform, or a mobile application) [82, 92–97]. Within this group, wearable devices were also utilised as either the primary delivery modality [98] or accompanying another delivery modality [57, 87, 88, 98]. Nine utilised *telehealth*, of which 8 delivered content over phone calls and 1 investigated SMS delivery [99], whereby participants were sent education material over text messages. Delivery modalities less frequently used included DVDs [100] and print [98, 101–103].

#### Intervention duration

The duration of the interventions ranged from 2 to 104 weeks (*M* = 20, *Mdn* = 12). 50.9% of the interventions were delivered over 12 weeks or less, with the most common intervention durations being 12 weeks (31.8%), 26 weeks (15.5%), and 52 weeks (17.3%).

#### Health behaviours targeted

##### Physical activity

Most included interventions addressed physical activity (*n* = 107, 93.9%). Twenty-two interventions targeted *aerobic activity* (e.g. walking, cycling) [28–30, 34, 43, 45, 50, 51, 57, 58, 61, 63, 75, 80, 81, 91, 104, 105]. Seven interventions focused on *resistance exercises* (e.g. lifting weights) [35, 40, 48, 67, 78]. Thirty-four interventions promoted a *combination* of aerobic and resistance exercises [27, 31–33, 36–39, 41, 42, 52, 53, 55, 59, 68, 76, 77, 79, 83, 85, 89, 90, 93, 101, 103, 106–108]. Four interventions practiced yoga [60, 62, 72] and one intervention [70] involved a combination of aerobic, resistance, and yoga exercises. Twenty-five interventions did not specify a particular exercise, instead focusing on increasing minutes of physical activity per week [46, 47, 54, 71, 74, 82, 86, 87, 94–100, 102, 109], reducing sedentary time [92], or a combination of these [69, 88, 110].

##### Nutrition

Thirty-five (30.7%) of the included interventions contained a nutritional component. Of these interventions, 12 focused on *diet restriction* through decreasing certain food groups consumed [56], or reducing total daily calorie intake [50]. Common recommendations for daily calorie intake in the included interventions were between 1200 and 2000 kcal/day [38, 58, 83] or reducing the participants current calorie intake by 600 kcal [85]. Comparatively, six interventions focused on *dietary change* and promoted increasing certain food groups [65, 84, 97], such as 5 servings of vegetables and 2 servings of fruit per day, and increasing intake of nuts, grains, and fish. Thirteen interventions utilised a *combination* of dietary restriction and dietary change strategies [34, 55, 59, 69, 79, 87, 105–108]. Two inventions cited a *particular diet plan*, such as an anti-inflammatory diet [73] or the Mediterranean diet [56]. Six interventions included *non-specified* dietary guidance or counselling [70, 74, 77, 95, 99, 111]. Three interventions included recommendations to *decrease alcohol consumption* [55, 85, 108].

##### Mental health

Overall, 19 of the 110 (17.3%) interventions featured a mental health component in their protocol. Six provided *mental health treatment* based on evidence based psychological therapies, such as cognitive behavioural therapy [45, 66, 95, 97, 105] or Mindfulness-Based Stress Reduction [79]. Seven interventions included *psycho-educational material* on social and emotional well-being [99], stress management [46, 56, 112], mindfulness [77], or psychological adjustment following a cancer diagnosis [111]. One intervention utilised meditation following a yoga session [60]. Three interventions described the use of ‘psychological support’ or counselling but did not provide further details [38, 42, 76].

### Meta-analysis of overall intervention effects

**Fig. 2:**
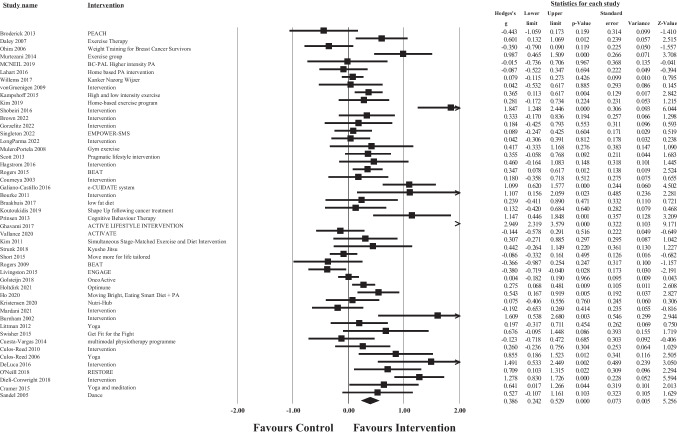
Forest plot of meta-analysis of effect sizes identified for each health behaviour intervention on post intervention Total QoL

**Fig. 3:**
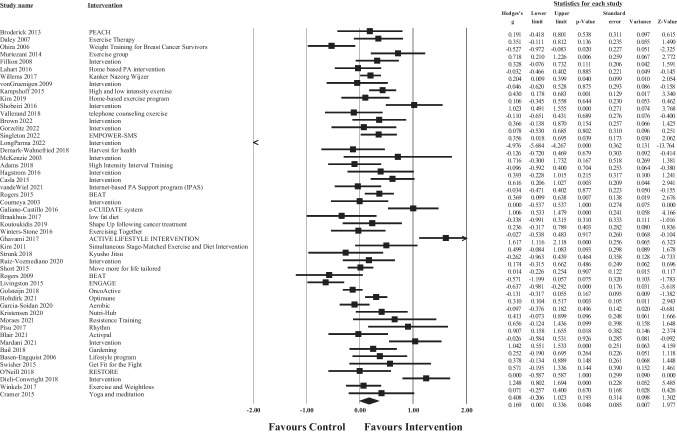
Forest plot of meta-analysis of effect sizes identified for each health behaviour intervention on post intervention physical well-being

**Fig. 4:**
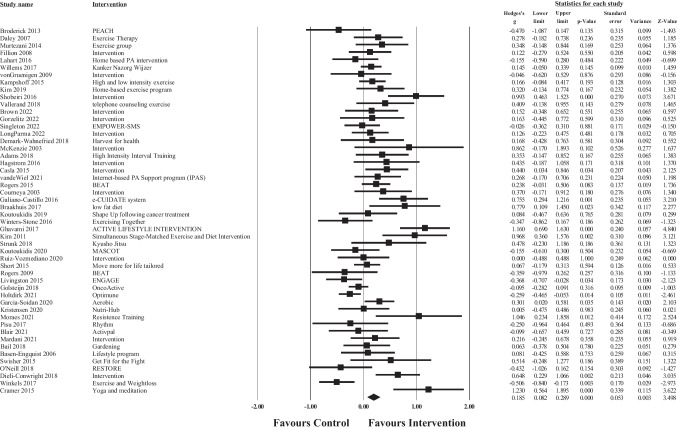
Forest plot of meta-analysis of effect sizes identified for each health behaviour intervention on post intervention emotional well-being

**Fig. 5:**
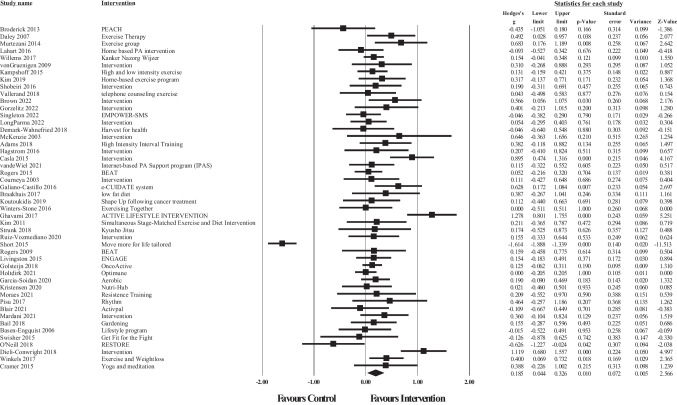
Forest plot of meta-analysis of effect sizes identified for each health behaviour intervention on post intervention social well-being

Post-treatment data was available for meta-analysis from 48 articles for total QoL (Fig. 2), 50 for physical well-being (Fig. 3), 50 for emotional well-being (Fig. 4), and 48 for social well-being (Fig. 5).

The overall pooled effect size of the interventions demonstrated a small significant, positive effect of healthy lifestyle interventions on cancer survivors’ total QoL (*g* = 0.32, 95% CI [0.17, 0.48], *p* >.001), physical well-being (*g* = 0.19, 95% CI [0.01, 0.36], *p* = 0.05), emotional well-being (*g* = 0.20, 95% CI [0.10, 0.31], *p* >.001), and social well-being (*g* = 0.18, 95% CI [0.05, 0.31], *p* = 0.01) in comparison to waitlist or usual care controls. For total QoL, 1 intervention demonstrated a negative effect, and favoured the control group over the intervention group [113]. Similar results were found for each of the subscale outcomes, whereby 3 interventions demonstrated negative effects (favouring the control condition) for physical well-being [73, 78, 113], 3 for emotional well-being [67, 95, 113, 114], and 2 for social well-being [103]. Consequently, these results should be interpreted with caution. According to Cohen’s criteria, substantial heterogeneity was observed for emotional well-being (*Q* = 142.99, *p* <.001; *I*^2^ = 65.73) and considerable heterogeneity was observed for total QoL (*Q* = 236.19, *p* <.001; *I*^2^ = 80.10), physical well-being (*Q* = 384.89, *p* <.001; *I*^2^ = 87.27), and social well-being (*Q* = 248.98, *p* <.001; *I*^2^ = 81.12); visual inspection of each forest plot demonstrates dispersion across 0.

### Subgroup analyses

Table 1 summarises the results of the pre-specified subgroup analyses conducted to examine differences arising from the inclusion of a mental health component, mode of delivery, and the duration of the intervention on each of the QoL outcomes.

**Table 1:** Prespecified and post hoc subgroup analyses

Meta-analysis	*N* interventions	Sub-group (*N* interventions)	*Hedge’s g* [95% CI]	Difference between subgroups: *Q*	Heterogeneity	
					*I*^*2*^	*Q*
Total QoL						
Mental health	48	Yes (12)	0.26 [0.10, 0.42]	2.05, df = 1, *p* = 0.15	43.08	19.33, df = 11, *p* = .06
		No (36)	0.41 [0.22, 0.60]		83.87	216.98, df = 35, *p* <.001
Mode of delivery	47	Individual (16)	0.65 [0.27, 1.03]	15.48, df = 5, *p =*.01*	87.42	119.27, df = 15, *p* <.001
		Group (20)	0.35 [0.14, 0.57]		71.28	66.15, df = 16, *p* <.001
		Digital (5)	0.26 [−0.02, 0.53]		79.58	19.59, df = 4, *p* <.001
		Telehealth (2)	0.14 [−0.15, 0.44]		0	0.41, df = 5, *p* = 0.52^†^
		Print (2)	−0.11 [−0.33, 0.11]		0	0.16, df = 1, *p* = 0.69^†^
		Multiple (2)	0.21 [−0.46, 0.88]		81.75	5.48, df = 1, *p* = 0.02
Duration	48	≤12 (29)	0.35 [0.18, 0.51]	0.44, df = 1, *p* = 0.50	73.88	107.18, df =28, *p* <.001
		≥13 (19)	0.45 [ 0.19, 0.71]		86.01	128.68, df =17, *p* <.001
Multicomponent	48	Yes (18)	0.50 [0.26, 0.74]	1.36, df = 1, *p* = 0.24	80.85	88.77, df = 17, *p* <.001
		No (30)	0.32 [0.14, 0.50]		79.84	143.88, df = 29, *p* <.001
Measure	43	FACT (26)	0.33 [0.16, 0.49]	0.93, df = 1, *p =* 0.33	64.44	70.30, df = 25, *p* <.001
		EORTC QLQ-C30 (17)	0.48 [0.20, 0.77]		88.92	144.39, df = 16, *p* <.001
Level of measure	48	Primary (18)	0.42 [0.21, 0.63]	0.16, df = 1, *p* = 0.69	76.63	72.73, df = 17, *p* <.001
		Secondary (30)	0.37 [0.17, 0.56]		82.00	161.07, df = 29, *p* <.001
Physical well-being						
Mental health	50	Yes (14)	0.22 [ 0.11, 0.34]	0.10, df = 1, *p* = 0.76	18.93	16.04, df = 13, *p* =0.25^†^
		No (36)	0.18 [−0.06, 0.42]		90.45	366.49, df = 34, *p*<.001
Mode of delivery	49	Individual (16)	0.36 [ 0.03, 0.68]	15.95, df = 4, *p* = 0.003*	83.93	93.31, df = 15, *p*<.001
		Group (22)	−0.03 [−0.36, 0.31]		91.30	241.28, df = 21, *p*<.001
		Digital (6)	0.20 [−0.06, 0.46]		80.01	25.01, df = 5, *p*<.001
		Telehealth (3)	0.27 [−0.05, 0.58]		26.95	2.74, df = 2, *p* = 0.26^†^
		Print (2)	0.51 [−0.50, 1.51]		92.64	13.58, df =1, *p*<.001
Duration	50	≤12 (27)	0.33 [0.18, 0.49]	46.73, df = 1, *p* = 0.03*	69.07	84.06, df = 26, *p*<.001
		≥13 (23)	−0.04 [−0.35, 0.26]		92.48	279.11, df = 22, *p*<.001
Multicomponent	50	Yes (23)	0.29 [0.16, 0.42]	1.87, df = 1, *p* = 0.17	52.59	46.40, df = 22 *p* = .002
		No (27)	0.07 [−0.22, 0.35]		91.96	323.57, df = 26, *p*<.001
Measure	55	FACT (17)	−0.07 [−0.52, 0.38]	3.72, df = 2, *p* = 0.16	93.44	243.93, df = 16, *p*<.001
		EORTC QLQ-C30 (16)	0.39 [0.13, 0.64]		85.67	104.71, df = 15, *p*<.001
		SF (15)	0.16 [0.01, 0.31]		32.64	20.78, df =14, *p*=0.11^†^
Level of measure	48	Primary (15)	0.31 [0.11, 0.52]	1.87, df =1, *p* = 0.17	73.75	53.33, df = 14, *p* <.001
		Secondary (35)	0.10 [−0.13,0 0.33]		89.57	326.11, df = 34, *p* <.001
Emotional well-being						
Mental health	50	Yes (14)	0.10 [−0.08, 0.36]	0.93, df = 1, *p* = 0.36	60.90	34.17, df = 13, *p =* .001
		No (36)	0.23 [0.10, 0.36]		67.68	106.06, df = 5, *p* <.001
Mode of delivery	49	Individual (17)	0.30 [0.08, 0.51]	3.27, df = 4, *p* = 0.51	66.27	47.44, df = 16, *p* <.001
		Group (21)	0.12 [−0.05, 0.28]		62.71	53.63, df = 20, *p* <.001
		Digital (6)	0.08 [−0.16, 0.32]		76.24	21.05, df = 5, *p* =.001
		Telehealth (3)	0.41 [−0.17, 0.98]		75.82	8.27, df = 2, *p* =.02
		Print (2)	0.10 [−0.12, 0.32]		53.74	2.16, df = 1, *p* = .14^†^
Duration	50	≤12 (27)	0.23 [0.08, 0.39]	0.84, df = 1, *p* = 0.36	68.45	82.42, df = 26, *p* <.001
		≥13 (23)	0.14 [−0.01, 0.28]		63.25	59.87, df = 22, *p* <.001
Multicomponent	50	Yes (23)	0.21 [0.04, 0.38]	0.13, df = 1, *p* = 0.72	71.92	78.36, df = 22, *p* <.001
		No (27)	0.17 [0.04, 0.30]		59.73	64.56, df = 26, *p* <.001
Measure	49	FACT (18)	0.22 [0.06, 0.37]	0.50, df = 2, *p* = 0.78	49.11	33.40, df = 17, *p* =.01
		EORTC QLQ-C30 (16)	0.23 [0.04, 0.43]		75.61	61.51, df = 15, *p* <.001
		SF (15)	0.14 [−0.05,0.33]		55.88	31.73, df = 14, *p* = .004
Level of measure	50	Primary (14)	0.33 [0.13, 0.53]	2.89, df = 1, *p* = 0.09	71.43	45.50, df = 13, *p* <.001
		Secondary (36)	0.13 [0.004, 0.25]		63.02	94.65, df = 35, *p* <.001
Social well-being						
Mental Health	48	Yes (13)	0.07 [−0.03, 0.17]	2.01, df = 1, *p* = 0.16	0	9.80, df = 12, *p* = 0.64^†^
		No (35)	0.23 [0.03, 0.43]		85.71	237.02, df = 34, *p* <.001
Mode of delivery	48	Individual (16)	0.40 [0.18, 0.62]	7.30, df = 4, *p* = 0.12	65.20	43.11, df = 15, *p* <.001
		Group (21)	0.16 [0.04, 0.28]		26.88	6.84, df = 20, *p* = .15^†^
		Digital (6)	0.13 [−0.01, 0.26]		56.97	11.62, df = 5, *p* = 0.02
		Telehealth (3)	0.03 [−0.23, 0.28]		0	0.58, df = 2, *p* = 0.75^†^
		Print (2)	−0.63 [−2.57, 1.30]		98.06	51.46, df = 1, *p* = 0.99^†^
Duration	48	≤12 (26)	0.15 [−0.10, 0.39]	0.35, df = 1, *p* = 0.56	86.89	190.68, df = 25, *p* <.001
		≥13 (22)	0.23 [0.09, 0.36]		57.19	49.05, df = 21, *p* <.001
Multicomponent	48	Yes (22)	0.21 [0.06, 0.35]	0.13, df = 1, *p* = 0.72	59.14	51.39, df = 21, *p* <.001
		No (26)	0.16 [−0.05, 0.37]		87.98	153.14, df = 25, *p* <.001
Measure	47	FACT (17)	0.14 [−0.24, 0.51]	0.25, df = 2, *p* = 0.88	91.05	178.68, df = 16, *p* <.001
		EORTC QLQ-C30 (16)	0.22 [0.07, 0.37]		67.31	45.89, df = 15, *p* <.001
		SF (14)	0.24 [0.09, 0.39]		22.69	16.82, df = 13, *p* = .21^†^
Level of measure	48	Primary (14)	0.24 [0.13, 0.36]	0.85, df = 1, *p* = 0.36	15.20	15.33, df = 13, *p* = 0.29^†^
		Secondary (34)	0.14 [−0.06, 0.33]		85.48	227.33, df = 33, *p* <.001

*The difference between groups is *p* <0.05

^†^Heterogeneity in this group is not significant

#### Mental health component

There were no significant differences in effect between interventions with or without a mental health component. Heterogeneity varied across these analyses: Heterogeneity was considerable on total QoL and emotional well-being subscales, whereas physical well-being and social well-being had no significant heterogeneity.

#### Modality

The mode of delivery subgroup analyses demonstrated a significant subgroup effect on total QoL and physical well-being. For total QoL, the individual (*g* = 0.65, 95% CI [0.27, 1.03]) and group modalities (*g* = 0.35, 95% CI [0.14, 0.57]) were associated with significant positive effects (favouring the intervention group). No other delivery modality was significant. Conversely, on the physical well-being outcome, only the individual modality (*g* = 0.36, 95% CI [0.03, 0.68]) was associated with a significant positive effect (favouring the intervention). However, these results should be interpreted with caution due to covariation distribution. Only two or three trials were included in the analysis for the print, telehealth, and multiple subgroups. Therefore, we cannot confidentially conclude that this is a true subgroup effect. Heterogeneity notably reduced in the group modality subgroup with the social well-being outcome and reduced in the smaller groups across the analyses, specifically the telephone and print subgroups.

#### Duration

There was a significant subgroup effect of duration on the physical well-being outcome. Shorter interventions (*g* = 0.33, 95% CI [0.18, 0.49]) were associated with a small positive effect and favoured the intervention group, whereas longer interventions (*g* = −0.04, 95% CI [−0.35, 0.26]) did not demonstrate a significant effect. However, substantial unexplained heterogeneity remained within each of the subgroups.

#### Sources of heterogeneity

The post hoc subgroup analyses exploring additional sources of heterogeneity are also presented in Table 1. None of the post hoc subgroup analyses identified significant associations across all outcomes. Heterogeneity remained considerable across these subgroup analyses, with the exception of studies which measured QoL as their primary outcome on the social well-being subscale (*I*^2^ = 15.20), and studies which used the SF to measure physical well-being (*I*^2^ = 32.64) and social well-being subscales (*I*^2^ = 22.69). 

### Narrative synthesis of interventions on QoL

Twenty-two studies investigating 31 interventions were excluded from the meta-analysis as they did not provide post-treatment means and standard deviations [36, 38, 43, 48, 51, 53, 59, 74, 76, 80, 86, 89–91, 98, 107–109, 111, 115, 116]. Total QoL was reported in 14 studies evaluating 19 interventions. Of these, 5 (26.3%) interventions demonstrated significant improvements compared to control [36, 38, 51, 76, 91]. For physical well-being, 10 of the 25 interventions (40%) reporting this outcome showed significant improvements compared to control [36, 51, 74, 91, 111, 115]. In terms of emotional well-being, 6 of the 24 interventions (25%) reported greater improvements in the intervention group [51, 76, 91, 115], though in one study [43] this benefit was only found in a subgroup of participants (those not currently taking endocrine therapy). Lastly, for social well-being, only 1 out of 25 interventions reported significant improvements compared to a waitlist intervention [111]. Moreover, Saarto and colleagues [80] found that an aerobic exercise intervention demonstrated significantly *less* change over time in social well-being compared to the usual care control group.

Three studies investigated 5 interventions with a mental health component, all of which showed significant improvements in at least one area of QoL. Three of the interventions utilised individual counselling and demonstrated significant improvements in total QoL [38, 76], physical well-being [42, 76], and emotional well-being [76] compared to the control groups. Naumann and colleagues [76] also investigated group counselling, which demonstrated significant improvements in physical well-being compared to the control group. Lastly, one intervention investigated by Chang and colleagues [111] involved an e-health booklet on psychological adjustment after cancer and this intervention demonstrated significant improvements in physical well-being and social well-being compared to the control group.

In terms of mode of delivery, all interventions that demonstrated significant improvements in all QoL measures utilised face-to-face delivery [individual *n* = 6, group *n* = 3; 36, 38, 51, 76, 91, 111], with the exception of one telehealth intervention implemented by Baruth and colleagues [115], which demonstrated significant improvements in physical well-being and emotional well-being in comparison to the control group.

Finally, with regard to duration, 17 interventions were offered over 12 weeks or less. Of these interventions, 4 (23.5%) demonstrated improvements in total Qol [36, 38, 51, 76], 7 (41.2%) demonstrated significant improvements in physical well-being [36, 42, 51, 76, 111, 115], 4 (23.5%) demonstrated significant improvements in emotional well-being [51, 76, 115], and 1 (5.8%) demonstrated significant improvements in social well-being [111] compared to the control group. Fourteen interventions were delivered over 13 weeks or more. Only 1 (7.1%) intervention demonstrated improvements in total QoL [91], 3 (21.4%) demonstrated improvements in physical well-being [59, 74, 91], and 1 (7.1%) demonstrated improvements in emotional well-being in comparison to the control group [91].

### Risk of bias

**Fig. 6:**
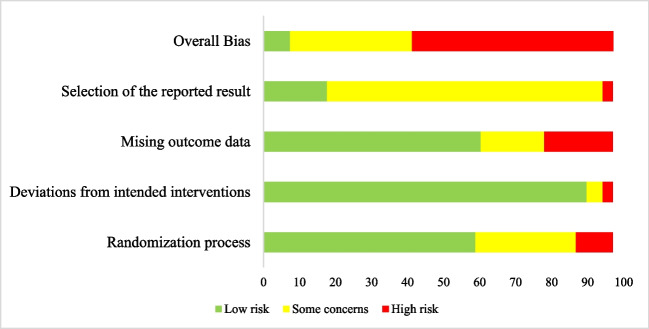
Risk of bias assessment for included domains as percentages across all studies included in the meta-analysis

The results from the risk of bias assessment are presented in Table 3 (Multimedia C) and a visual representation is provided in Fig. 6. Overall, the risk of bias was high for 55.9% of articles included in the meta-analysis. Domain 5, selection of the reported result, was the biggest contributor for risk of bias concerns, as most of the studies did not publish prespecified measurements or a data analysis plan. Consequently, only 5 studies were rated as having low risk of bias.

### Publication bias

Publication bias was indicated by the Egger’s regression intercept for the Total QoL outcome, 1.90, 95% CI [0.40, 3.40], *p* = .01, and the emotional well-being subscale, 1.92, 95% CI [0.09, 3.75], *p* = .04.

## Discussion

This systematic review and meta-analysis updates and extends the current evidence for the use of healthy lifestyle interventions to improve the QoL in post-treatment cancer survivors. Overall, results from the meta-analysis indicate a small but significant effect in favour of healthy lifestyle interventions’ positive impact on total QoL and on the dimensions of physical well-being, emotional well-being, and social well-being compared to a usual care or waitlist control. However, there was notable heterogeneity among the included studies and the majority did not find a significant effect of the intervention on all QoL outcomes. This finding was corroborated by studies included in the narrative synthesis, where out of 22 healthy lifestyle interventions examined, 17 did not differ from the usual care or waitlist control groups in each of the QoL domains. The observed heterogeneity in the results aligns with the inconsistencies found in previous research on this topic.

A unique contribution of this paper was to investigate whether the association between the intervention and QoL is moderated by key intervention characteristics, primarily the inclusion of a mental health component. There was no evidence that the inclusion of a mental health component impacted the association between participation in a healthy lifestyle intervention and QoL. Consequently, there is a discrepancy between what cancer survivors request to be part of a healthy lifestyle program and support from current research on these interventions impact on QoL. A potential explanation is that improving physical well-being through physical activity and diet also addresses emotional well-being and overall QoL [117]. However, it is premature to discount the usefulness of including a mental health component, given the small number of studies which continued to display high levels of heterogeneity. Consequently, more evidence is required to appropriately answer this question. Alternatively, including a mental health component may have benefits in other areas, such as addressing barriers experienced by cancer survivors in participating in physical activity and a nutritious diet [18, 19]. Furthermore, psychosocial issues are one of the most prominent unmet needs described by cancer survivors [118] and including a component addressing these has the potential to make cancer survivors feel more supported following treatment. Therefore, future reviews might consider investigating whether including a mental health component in a healthy lifestyle intervention is associated with increased physical activity and diet outcomes or promotes more positive qualitative feedback compared to interventions which do not.

In contrast, mode of delivery and intervention duration emerged as predictors of intervention efficacy: Face-to-face delivery, either individually or in a group format, was associated with significantly higher total QoL. Individual face-to-face delivery was also associated with significantly higher physical well-being. Similarly, shorter interventions were associated with greater improvements in physical well-being. This finding aligns to some extent with the findings from a meta-analysis completed by Ferrer and colleagues [7], which investigated exercise interventions for cancer survivors and also found that intervention duration was inversely associated with QoL outcomes. However, Ferrer and colleagues found one exception to this relationship where the *intensity* of the intervention moderated outcomes, such that longer interventions (i.e. 26 weeks) with *higher* intensity exercise were associated with greater changes in QoL than shorter interventions (i.e. 8 weeks) and/or interventions with lower intensity exercise. Thus, while select longer interventions may be beneficial, collectively the weight of evidence from both prior and current meta-analyses support the implementation of short-term and face-to-face delivered healthy lifestyle interventions at the completion of cancer treatment, particularly for those looking to improve their physical well-being.

Nagpal and colleagues [119] have previously recommended that adherence is an important consideration when evaluating the efficacy of exercise interventions, due to the implications on whether participants receive the recommended ‘dose.’ Shorter durations and face-to-face modalities may promote greater engagement and adherence by minimising time commitments and enhancing accountability [120]. Further, interventions involving intense exercise may necessitate supervision to ensure participant safety and offer the advantage of increased accountability and tailoring. However, adherence data was not extracted in either the current study, nor the meta-analysis conducted by Ferrer and colleagues. To date, no research has directly compared the degree of adherence to shorter verses longer healthy lifestyle interventions in the cancer survivor or other relevant populations, such as older individuals or individuals with other chronic health conditions. Consequently, future primary research should consider comparing the same healthy lifestyle interventions with differing durations or delivery modalities to investigate adherence and its relationship to QoL outcomes. Future reviews should consider extracting adherence data to investigate its relationship with other intervention characteristics and outcomes. This meta-analysis provides preliminary evidence to suggest that interventions delivered via telephone or online can lead to comparable outcomes to face-to-face interventions; however, more studies are required to compare the different delivery modalities on QoL in cancer survivors.

### Limitations

Although the overall meta-analysis and subgroup analyses yielded significant findings, these results should be interpreted with caution due to high levels of heterogeneity, limited power, high risk of bias, and lack of follow-up data. High levels of heterogeneity are commonly reported in meta-analyses on this topic. Notable heterogeneity continued across the pre-defined subgroup analyses, with only a reduction observed in individual subgroups, typically characterised by a low number of included studies (i.e. fewer than 10 studies). Additionally, the current meta-analysis may have limited power to detect an effect of the healthy lifestyle interventions on QoL, as less than one third of the included studies were designed to measure QoL. Consequently, the majority of the included studies may not be adequately powered to detect an effect on QoL. We attempted to address these limitations through post hoc subgroup analyses investigating multi-verse single-component interventions, whether QoL was measured as a primary or secondary outcome, and the type of outcome used, however, nil differences or reductions in heterogeneity were observed. Additionally, the validity of the results may be impacted by the quality of the studies, as the majority of them presented with a high risk of bias. Finally, as this current meta-analysis did not extract follow-up data, we are unable to evaluate whether the effects on QoL are maintained after the intervention period.

Additionally, there may be clinical factors that may moderate the efficacy of healthy lifestyle interventions on QoL in cancer survivors that were not explored in this study. A recent follow-up analysis conducted by Schleicher and colleagues [121] identified that breast cancer survivors participating the BEAT intervention who had a longer time since diagnosis (<24 months) and those who did not have a history of chemotherapy demonstrated greater increases in QoL. Schleicher and colleagues suggested that this may be due to perceived physical functioning, as cancer survivors with a more recent diagnosis may be experiencing acute side effects from treatment, such as fatigue and nausea. This finding was particularly relevant for time since diagnosis, as those who were more than 24 months post treatment were also more likely to engage in more moderate and vigorous physical activity post treatment. Future systematic reviews and meta-analyses should consider extracting data on time since diagnosis and treatment type to explore these as potential moderating factors.

## Conclusion

Overall, the current meta-analysis suggests that participating in any healthy lifestyle intervention following cancer treatment is likely to have positive benefits on QoL. Interventions which are delivered face-to-face or over a shorter duration may have a greater impact on the efficacy of such interventions; however, only a few randomised control trials have investigated alternative delivery modalities, such as digital or telehealth. Furthermore, few randomised control trials have specifically investigated the inclusion of a mental health component to healthy lifestyle interventions. Consequently, there is a need for future research to develop and rigorously evaluate healthy lifestyle interventions which also address mental health and utilise alternative delivery modalities.

## Supplementary information

ESM 1 (DOCX 24 kb)

ESM 2 (DOCX 154 kb)

ESM 3 (DOCX 350 kb)

## Data Availability

The datasets generated and analysed during the current study are available from the corresponding author on reasonable request.
