# Supplementary material for: A meta-analysis of healthy lifestyle interventions addressing quality of life of cancer survivors in the post treatment phase
Source: J Cancer Surviv. 2024 Jan 11;19(3):940–56. doi: 10.1007/s11764-023-01514-x (PMC12081566; doi:10.1007/s11764-023-01514-x)
Supplement: Supplementary file 1 — (DOCX 24 kb) [file 11764_2023_1514_MOESM1_ESM.docx]

**Multimedia A - A meta-analysis of healthy lifestyle interventions addressing quality of life of cancer survivors in the post treatment phase.**

**Scopus**

( TITLE-ABS ( ( cancer* OR tumor* OR tumour* OR neoplas* OR malignan* OR carcinoma* OR adenocarcinoma* OR choriocarcinoma* OR leukemia* OR leukaemia* OR metastat* OR sarcoma* OR teratoma* ) AND ( survivor* OR survival ) ) AND TITLE-ABS ( ( "healthy lifestyle" OR "healthy living" OR lifestyle OR behavio?r OR "lifestyle changes" OR "health behavio?r" OR sedentary* ) OR ( "Physical activity" OR pa OR exercise OR "active living" OR "activity level" OR "physical fitness" OR "physical endurance" ) OR ( nutrition* OR diet* OR food* OR "food intake" ) OR ( weight* OR "weight control" OR "body weight" OR "weight loss" OR "weight management" OR "body mass index" OR bmi ) ) AND TITLE-ABS ( random* OR rct OR "clinical trial*" OR trial* OR intervention OR program OR therapy ) AND ALL ( "quality of life*" OR qol OR "health related quality of life" OR "health-related quality of life" OR hrqol ) AND NOT INDEX ( medline ) )

**Medline**

1. exp neoplasms/

2. (cancer* or tumor* or tumour* or neoplas* or malignan* or carcinoma* or adenocarcinoma* or choriocarcinoma* or leukemia* or leukaemia* or metastat* or sarcoma* or teratoma*).ab,ti.

3. 1 or 2

4. exp survivors/ or exp cancer survivor/

5. (survivor* or survival).ab,ti.

6. 4 or 5

7. 3 and 6

8. life style/ or health behavior/ or Sedentary Behavior/

9. (health* or lifestyle* or "life style*" or behavio?r or "active living" or "health behavio?r" or sedentary*).ab,ti.

10. exp exercise/ or exp physical fitness/ or physical endurance/ or exp walking/ or resistance training/

11. ("Physical activity" or PA or exercise or "active living" or "activity level" or "physical fitness" or "physical endurance" or strength* or walk* or "resistance training" or aerobic*).ab,ti.

12. nutritional assessment/ or nutritional status/ or diets/ or food/

13. (nutrition* or diet* or food*).ab,ti.

14. body weight/ or weight loss/ or body mass index/

15. (weight* or "body weight" or "weight loss" or "weight management" or "weight control" or "body mass index" or BMI).ab,ti.

16. or/8-15

17. exp Randomized Controlled Trial/ or random allocation/

18. (random* or RCT or trial* or "clinical trial" or intervention or program or therapy).ab,ti.

19. 17 or 18

20. exp "Quality of Life"/

21. ("quality of life*" or QoL or QOL or "health related quality of life" or "health-related quality of life" or HRQoL or HRQOL).tw,kw.

22. 20 or 21

23. 7 and 16 and 19 and 22

24. limit 23 to humans

**PsycINFO**

1. exp neoplasms/

2. (cancer* or tumor* or tumour* or neoplas* or malignan* or carcinoma* or adenocarcinoma* or choriocarcinoma* or leukemia* or leukaemia* or metastat* or sarcoma* or teratoma*).ab,ti.

3. 1 or 2

4. exp survivors/

5. (survivor* or survival).ab,ti.

6. 4 or 5

7. 3 and 6

8. lifestyle/ or active living/ or lifestyle changes/ or health behavior/ or sedentary behavior/

9. ("healthy lifestyle" or "healthy living" or lifestyle* or "life style*" or “active living” or "lifestyle changes" or "health behavio?r" or sedentary*).ab,ti.

10. exp physical activity/ or exp exercise/ or active living/ or activity level/ or exp physical fitness/ or physical endurance/ or walking/ or physical strength/ or exp aerobic exercise/

11. ("Physical activity" or PA or exercise or "active living" or "activity level" or "physical fitness" or "physical endurance" or strength* or walk* or "resistance training" or aerobic*).ab,ti.

12. nutrition/ or diets/ or food intake/ or food/

13. (nutrition* or diet* or food* or "food intake").ab,ti.

14. weight control/ or body weight/ or weight loss/ or body mass index/

15. (weight* or "weight control" or "body weight" or "weight loss" or "weight management" or "body mass index" or BMI).ab,ti.

16. or/8-15

17. exp Intervention/

18. (random* or RCT or trial* or intervention or program or therapy).ab,ti.

19. 17 or 18

20. 7 and 16 and 19

21. exp "Quality of Life"/

22. ("quality of life*" or QoL or QOL or "health related quality of life" or "health-related quality of life" or HRQoL or HRQOL).tw,id.

23. 20 and 22

**CINHAL**

S1 (MH "Neoplasms+")

S2 TI (cancer* OR tumor* OR tumour* OR neoplas* OR malignan* OR carcinoma* OR adenocarcinoma* OR choriocarcinoma* OR leukemia* OR leukaemia* OR metastat* OR sarcoma* OR teratoma*) OR AB (cancer* or tumor* or tumour* OR neoplas* OR malignan* OR carcinoma* OR adenocarcinoma* OR choriocarcinoma* OR leukemia* OR leukaemia* OR metastat* OR sarcoma* OR teratoma*)

S3 (MH "Survivors+") OR (MH "Cancer Survivors")

S4 TI(survivor* OR survival OR survivorship) OR AB(survivor* OR survival OR survivorship)

S5 S1 OR S2

S6 S3 OR S4

S7 S5 AND S6

S8 (MH "Life Style Changes") OR (MH "Life Style+") OR (MH "Behavioral Changes") OR (MH "Life Style, Sedentary+")

S9 TI("healthy lifestyle" OR "healthy living" OR lifestyle OR active living OR "lifestyle changes" OR "health behavio?r" OR sedentary*) OR AB("healthy lifestyle" OR "healthy living" OR lifestyle OR active living OR "lifestyle changes" OR "health behavio?r" OR sedentary*)

S10 (MH "Exercise+") OR (MH "Physical Activity+") OR (MH "Physical Fitness") OR (MH "Aerobic Exercise+") OR (MH "Resistance Training")

S11 TI("Physical activity" OR PA OR exercise OR "active living" OR "activity level" OR "physical fitness" OR "physical endurance" OR strength* OR walk* OR "resistance training" OR aerobic*) OR AB("Physical activity" OR PA OR exercise OR "active living" OR "activity level" OR "physical fitness" OR "physical endurance" OR strength* OR walk* OR "resistance training" OR aerobic*)

S12 (MH "Nutrition+") OR (MH "Nutritive Value+") OR (MH "Food Intake+")

S13 TI(nutrition* OR diet* OR food* OR "food intake") OR AB(nutrition* OR diet* OR food* OR "food intake")

S14 (MH "Weight Reduction Programs") OR (MH "Body Weights and Measures+") OR (MH "Body Weight Changes+") OR (MH "Body Weight+") OR (MH "Weight Control") OR (MH "Weight Loss") or (MH "Body Mass Index")

S15 TI(weight* OR "weight control" OR "body weight" OR "weight loss" OR "weight management" OR "body mass index" OR BMI) OR AB(weight* OR "weight control" OR "body weight" OR "weight loss" OR "weight management" OR "body mass index" OR BMI)

S16 S8 OR S9 OR S10 OR S11 OR S12 OR S13 OR S14 OR S15

S17 (MH "Randomized Controlled Trials+") OR (MH "Clinical Trials+")

S18 TI(random* OR RCT OR trial* OR "clinical trial" OR intervention OR program OR therapy) OR AB(random* OR RCT OR trial* OR "clinical trial" OR intervention OR program OR therapy)

S19 S17 OR S18

S20 S7 AND S16 AND S19

S21 (MH "Quality of Life+")

S22 TW("quality of life" or QoL or QOL or "health related quality of life" or "health-related quality of life" or HRQoL or HRQOL)

S23 S21 OR S22

S24 S20 AND S23

**Google scholar**

(Cancer survivor) AND (health* OR lifestyle*) OR ("physical activity" OR exercise OR training) OR (diet OR nutrition OR food) OR (weight or body) AND (random* OR trial* OR intervention OR program) AND (“quality of life”)

Cancer survivor AND Random* health OR lifestyle OR "physical activity" OR exercise OR training OR diet OR nutrition OR food OR weight OR body "quality of life "

Cancer survivor random health OR lifestyle OR "physical activity" OR exercise OR training OR diet OR nutrition OR food OR weight OR body "quality of life"

*First 200 references as per recommendations from Bramer, Rethlesfen, Kleijnen & Franco (2017)*
