# Supplementary material for: A meta-analysis of healthy lifestyle interventions addressing quality of life of cancer survivors in the post treatment phase
Source: J Cancer Surviv. 2024 Jan 11;19(3):940–56. doi: 10.1007/s11764-023-01514-x (PMC12081566; doi:10.1007/s11764-023-01514-x)
Supplement: Supplementary file 2 — (DOCX 154 kb) [file 11764_2023_1514_MOESM2_ESM.docx]

| Multimedia B - A meta-analysis of healthy lifestyle interventions addressing quality of life of cancer survivors in the post treatment phase.  Supplementary table  *Characteristics of included studies* | | | | | | | | | | | |
| --- | --- | --- | --- | --- | --- | --- | --- | --- | --- | --- | --- |
| Study | Population | Intervention and control | Post-treatment *N* | Mode of delivery | Duration (weeks) | Intervention components | | | QoL measure | QoL Life primary outcome? | QoL findings |
|  |  |  |  |  |  | Physical Activity | Nutrition | Mental Health |  |  |  |
| Adams et al. (2018) | Cancer type: Testicular  M^age^: 43.7  M^Months since diagnosis^: 96  Gender: 100% male | I: High Intensity Interval Training (HITT)  C: Usual care | I:29  C:13 | Individual | 12 | ✓ |  |  | SF-36 | No | **Total:** N/A  **Physical:** NS  **Emotional:** NS  **Social:** I>C |
| Alibhai et al. (2014) | Cancer type: Acute myeloid leukemia  M^age^: 56.1  M^Months since diagnosis^: 23.4  Gender: 55.3% female | I: Intervention  C: Waitlist | I:19  C:17 | Group | 12 | ✓ |  |  | EORTC QLQ-C30 | No | Compared mean change.  **Total:** NS  **Physical:** NS  **Emotional:** NS  **Social:** NS |
| Bail et al. (2018) | Cancer type: Breast  M^age^: 60.5  M^Months since diagnosis^: 64.8  Gender: 100% female | I: Gardening  C: Waitlist | I:19  C:17 | Individual and print | 52 | ✓ | ✓ |  | SF-36 | No | **Total:** N/A  **Physical:** NS  **Emotional:** NS  **Social:** NS |
| Baruth et al. (2015) | Cancer type: Breast  M^age^: 56.5  M^Months since diagnosis^: 5.17  Gender: 100% female | I: Home based walking  C: Waitlist | I:18  C:12 | Telephone and Pedometer | 12 | ✓ |  |  | SF-36 | Yes | **Total:** N/A  **Physical:** I>C  **Emotional:** I>C  **Social:** NS |
| Basen-Engquist et al. (2006) | Cancer type: Breast  M^age^: 55.1  M^Months since diagnosis^: 38.3  Gender: 100% female | I: Lifestyle program  C: Usual care | I:28  C:23 | Group | 24 | ✓ |  |  | Unknown | No | **Total:** N/A  **Physical:** NS  **Emotional:** NS  **Social:** NS |
| Blair et al. (2021) | Cancer type: breast, prostate, bladder, cervical, colon, endometrium, kidney, lymphoma, melanoma  M^age^: 69.6  M^Months since diagnosis^: 52.8  Gender: 66% female | I^1^: Activpal  I^2^: Activpal + health coaching  C: Waitlist | I^1^: 18  I^2^: 17  C: 18 | I^1^: Mobile application  I^2^: Mobile Application and telephone | 13 | ✓  ✓ |  |  | SF-36 | No | Compared mean change.  **Total:** N/A  **Physical:** NS  **Emotional:** NS  **Social:** NS |
| Bourke et al. (2011) | Cancer type: Colon  M^age^: 56.1  M^Months since diagnosis^: N/R  Gender: 33.3% female | I: Intervention  C: Usual care | I:8  C:9 | Group and print | 12 | ✓ | ✓ |  | FACT-C | No | **Total:** I>C (NS when comparing change over time)  **Physical:** N/R  **Emotional:** N/R  **Social:** N/R |
| Braakhuis et al. (2017) | Cancer type: Breast  M^age^: 55.5  M^Months since diagnosis^: N/R  Gender: 100% female | I^1^:Mediterranean diet  I^2^: low fat diet  C: Usual care | I^1^: 15  I^2^: 12  C: 13 | Group and print | 26 |  | ✓  ✓ | ✓  ✓ | FACT-G | No | **Total:** NS  **Physical:** NS  **Emotional:** NS  **Social:** NS |
| Broderick et al. (2013) | Cancer type: Breast, Colon, Lymphoma, and Oesophageal.  M^age^: 51.0  M^Months since diagnosis^: 9.1  Gender: 86% female | I: Prescribed Exercise After Chemotherapy (PEACH)  C: Usual Care | I:21  C:19 | Group | 8 | ✓ |  |  | FACT-G | No | Compared mean change.  **Total:** NS  **Physical:** I>C  **Emotional:** NS  **Social:** NS |
| Brown et al. (2018) | Cancer type: Colon  M^age^: N/R  M^Months since diagnosis^: N/R  Gender: 62% female | I^1^: COURAGE Low dose  I^2^: COURAGE High dose  C: Usual care | I^1^: 14  I^2^: 12  C: 13 | Individual, telephone, and email | 26 | ✓  ✓ |  |  | FACT-C | Yes | **Total:** I>C  **Physical:** I>C  **Emotional:** I>C  **Social:** NS |
| Brown et al. (2021) | Cancer type: Breast  M^age^: 59.4  M^Months since diagnosis^: 92  Gender: 100% female | I^1^: Exercise  I^2^: Diet  I^3^: Exercise + diet  C: Waitlist | I^1^: 62  I^2^: 56  I^3^: 66  C: 60 | Group | 52 | ✓  ✓ | ✓  ✓ |  | SF-36 | No | **Total:** N/A  **Physical:** I^3^>C  **Emotional:** NS  **Social:** NS |
| Brown et al. (2022) | Cancer type: Breast, Gynecologic, Hematologic, Genitourinary  M^age^: 58.0  M^Months since diagnosis^: 40.0  Gender: 86% female | I: Lifestyle Intervention  C: Waitlist | I:21  C:19 | Group | 15 | ✓ | ✓ |  | EORTC QLQ-C30 | Yes | **Total:** NS  **Physical:** I>C  **Emotional:** NS  **Social:** NS |
| Burnham and Wilcox (2002) | Cancer type: Breast and Colon  M^age^: 53.6  M^Months since diagnosis^: 9.7  Gender: 83.3% female | I: Intervention  C: Waitlist | I:12  C:6 | Individual | 10 | ✓ |  |  | Quality of Life index for cancer patients | No | **Total:** I>C  **Physical:** N/A  **Emotional:** N/A  **Social:** N/A |
| Casla et al. (2015) | Cancer type: Breast  M^age^: 49.1  M^Months since diagnosis^: 10.4  Gender: 100% female | I: Intervention  C: Waitlist | I:12  C:6 | Individual | 12 | ✓ |  |  | SF-36 | No | **Total:** N/A  **Physical:** I>C  **Emotional:** I>C  **Social:** I>C |
| Chang et al. (2020) | Cancer type: Esophageal  M^age^: 56.0  M^Months since diagnosis^: N/R  Gender: 9.1% female | I: Intervention  C: Waitlist | I:41  C:43 | Individual, smartwatch and print | 12 | ✓ | ✓ | ✓ | EORTC QLQ-C30 | Yes | Compared mean change.  **Total:** N/A  **Physical:** I>C  **Emotional:** NS  **Social:** I>C |
| Courneya et al. (2003) | Cancer type: Breast  M^age^: 59.0  M^Months since diagnosis^: N/R  Gender: 100% female | I: Intervention  C: Waitlist | I:24  C:28 | Individual | 15 | ✓ |  |  | FACT-G | Yes | **Total:** NS (I>C when comparing mean change)  **Physical:** NS (I>C when comparing mean change)  **Emotional:** NS  **Social:** NS |
| Cramer et al. (2015) | Cancer type: Breast  M^age^: 49.2  M^Months since diagnosis^: 30.3  Gender: 100% female | I: Yoga and meditation  C: Usual care | I:19  C:21 | Group | 12 | ✓ |  | ✓ | FACT-B | Yes | **Total:** I>C  **Physical:** NS  **Emotional:** I>C  **Social:** I>C |
| Cuesta-Vargas et al. (2014) | Cancer type: Breast  M^age^: 47.9  M^Months since diagnosis^: N/R  Gender: 100% female | I: Multimodal physiotherapy programme  C: Usual care | I:20  C:22 | Group | 8 | ✓ |  |  | EuroQoL-5D | No | **Total:** NS  **Physical:** N/A  **Emotional:** N/A  **Social:** N/A |
| Culos-Reed et al. (2006) | Cancer type: Breast  M^age^: 50  M^Months since diagnosis^: 56  Gender: 95% female | I: Yoga  C: Waitlist | I:18  C:18 | Group | 7 | ✓ |  |  | EORTC QLQ-C30 | Yes | **Total:** I>C  **Physical:** N/R  **Emotional:** I>C  **Social:** N/R |
| Culos-Reed et al. (2010) | Cancer type: Prostate  M^age^: 67.6  M^Months since diagnosis^: N/R  Gender: 100% male | I: Intervention  C: Waitlist | I:40  C:25 | Individual | 6 | ✓ |  |  | EORTC QLQ-C30 | No | **Total:** NS  **Physical:** N/R  **Emotional:** N/R  **Social:** N/R |
| Daley et al. (2007) | Cancer type: Breast  M^age^: 51.3  M ^Months since diagnosis^: NR  Gender: 100% female | I: Exercise Therapy  C: Usual Care | I: 33  C: 33 | Group | 8 | ✓ |  |  | FACT-G | Yes | **Total:** I>C  **Physical:** NS  **Emotional:** NS  **Social:** I>C |
| De Luca et al. (2016) | Cancer type: Breast  M^age^: 45.6  M ^Months since diagnosis^: 12.5  Gender: 100% female | I: Exercise Therapy  C: Usual care | I: 10  C: 10 | Individual | 24 | ✓ |  |  | FACT-G | No | **Total:** I>C  **Physical:** N/R  **Emotional:** N/R  **Social:** N/R |
| Demark-Wahnefried et al. (2018) | Cancer type: Breast  M^age^: 70.1  M ^Months since diagnosis^: 80.4  Gender: 100% female | I: Harvest for Health  C: Waitlist | I: 22  C: 20 | Group, email, and telephone | 52 | ✓ | ✓ |  | SF-36 | No | **Total:** N/A  **Physical:** NS  **Emotional:** I>C  **Social:** NS |
| Dieli-Conwright et al. (2018) | Cancer type: Breast  M^age^: 53.5  M ^Months since diagnosis^: 6.2  Gender: 100% female | I: Intervention  C: Usual care | I: 46  C: 45 | Individual | 16 | ✓ |  |  | FACT-G | No | **Total:** I>C  **Physical:** I>C  **Emotional:** I>C  **Social:** I>C |
| Fillion et al. (2008) | Cancer type: Breast  M^age^: 52.5  M ^Months since diagnosis^: NR  Gender: 100% female | I: Lifestyle intervention  C: Usual care | I: 44  C: 43 | Group | 4 | ✓ |  | ✓ | SF-12 | No | **Total:** N/R  **Physical:** N/R  **Emotional:** N/R  **Social:** N/R  **Other:**  Physical Composite: NS  Mental composite: NS |
| Galiano-Castillo et al. (2016) | Cancer type: Breast  M^age^: 48.3  M ^Months since diagnosis^: NR  Gender: 100% female | I: e-CUIDATE system  C: Usual care | I: 39  C: 37 | Digital | 8 | ✓ |  |  | EORTC QLQ-C30 | Yes | **Total:** I>C  **Physical:** I>C  **Emotional:** NS  **Social:** NS |
| Garcia-Soidan et al. (2020) | Cancer type: Breast  M^age^: 63.0  M^Months since diagnosis^: N/R  Gender: 100% female | I^1^: Strength  I^2^: Aquatic  I^3^: Aerobic  C: Usual care | I^1^: 74  I^2^: 65  I^3^: 79  C: 63 | Group | 104 | ✓  ✓  ✓ |  |  | SF-12 | Yes | **Total:** N/A  **Physical:** NS  **Emotional:** I^1^>C, I^2^>C, C>I^3^  I^1^>I^2^, I^1^>I^3^, I^2^>I^3^  **Social:** I^1^>C, I^2^>C, I^3^>C  I^1^>I^2^, I^3^>I^2^ |
| Ghavami and Akyolcu (2017) | Cancer type: Breast  M^age^: 49.0  M ^Months since diagnosis^: N/R  Gender: 100% female | I: Active Lifestyle Intervention  C: Usual care | I: 40  C: 40 | Individual | 24 | ✓ | ✓ |  | EORTC QLQ-C30 | No | **Total:** I>C  **Physical:** I>C  **Emotional:** I>C  **Social:** I>C |
| Golsteijn et al. (2018) | Cancer type: Prostate and Colorectal  M^age^: 66.5  M ^Months since diagnosis^: N/R  Gender: 13% female | I: OncoActive  C: Waitlist | I: 229  C: 222 | Online | 16 | ✓ |  |  | EORTC QLQ-C30 | No | **Total:** NS  **Physical:** I>C  **Emotional:** N/R  **Social:** N/R |
| Gorzelitz et al. (2022) | Cancer type: Endometrial  M^age^: 60.9  M ^Months since diagnosis^: 34.8  Gender: 100% female | I: Lifestyle intervention  C: Waitlist | I: 64  C: 71 | Face-to-face and youtube videos | 10 | ✓ |  |  | FACT-EN | No | **Total:** NS  **Physical:** NS  **Emotional:** NS  **Social:** NS |
| Hagstrom et al. (2016) | Cancer type: Breast  M^age^: 51.9  M ^Months since diagnosis^: 11.6  Gender: 100% female | I: Lifestyle intervention  C: Usual care | I: 19  C: 20 | Individual | 16 | ✓ |  |  | FACT-G | No | **Total:** I>C  **Physical:** I>C  **Emotional:** NS  **Social:** NS |
| Herrero et al. (2006) | Cancer type: Breast  M^age^: 50.5  M ^Months since diagnosis^: 35.9  Gender: 100% female | I: Lifestyle intervention  C: Usual care | I: 8  C: 8 | Individual | 8 | ✓ |  |  | EORTC QLQ-C30 | Yes | Compared mean change.  **Total:** I>C  **Physical:** I>C  **Emotional:** N/R  **Social:** N/R |
| Ho et al. (2020) | Cancer type: Colorectal  M^age^: 65.2  M^Months since diagnosis^: N/R  Gender: 36.8% female | I^1^: Moving Bright, Eating Smart Diet + PA  I^2^: Moving Bright, Eating Smart Diet  I^3^: Moving Bright, Eating Smart PA  C: Usual care | I^1^: 55  I^2^: 56  I^3^: 56  C: 56 | Individual, pedometer and telephone | 52 | ✓  ✓ | ✓  ✓ |  | FACT-G | Yes | **Total:** I^1^>C  **Physical:** N/R  **Emotional:** N/R  **Social:** N/R |
| Holtdirk et al. (2021) | Cancer type: Breast  M^age^: 49.9  M ^Months since diagnosis^: N/R  Gender: 100% female | I: Optimune  C: Usual care | I: 141  C: 165 | Online | 12 | ✓ | ✓ | ✓ | WHOQOL-BREF | Yes | **Total:** I>C  **Physical:** I>C  **Emotional:** I>C  **Social:** NS |
| Kampshoff et al. (2015) | Cancer type: Breast, Colon, Ovarian, Lymphoma, Cervix, Testis  M^age^: 53.7  M ^Months since diagnosis^: N/R  Gender: 80% female | I^1^: High Intensity Exercise  I^2^: Low to Moderate Intensity Exercise  C: Waitlist | I^1^: 91  I^2^: 95  C: 91 | Group | 12 | ✓ |  |  | EORTC QLQ-C30 | No | **Total:** I^1^>C  **Physical:** I^1^>C, I^2^>C  **Emotional:** NS  **Social:** NS |
| Kim et al. (2011) | Cancer type: Breast  M^age^: 45.8  M ^Months since diagnosis^: 12.7  Gender: 100% female | I: Simultaneous Stage-Matched Exercise and Diet Intervention  C: Usual care | I: 23  C: 22 | Telephone and print | 12 | ✓ | ✓ |  | EORTC QLQ-C30 | No | Compared mean change.  **Total:** NS  **Physical:** NS  **Emotional:** NS  **Social:** NS |
| Kim et al. (2019) | Cancer type: Colorectal  M^age^: 56.2  M ^Months since diagnosis^: 10.7  Gender: 100% female | I: Home-based exercise program  C: Usual care | I: 30  C: 28 | DVDs | 12 | ✓ |  |  | FACT-C | Yes | Compared mean change.  **Total:** NS  **Physical:** NS  **Emotional:** NS  **Social:** NS |
| Koutoukidis et al. (2019) | Cancer type: Endometrial  M^age^: 62.1  M ^Months since diagnosis^: 14.4  Gender: 100% female | I: Shape Up following cancer treatment  C: Usual care | I: 25  C: 24 | Groups | 8 | ✓ | ✓ |  | EORTC QLQ-C30 | No | Compared mean change.  **Total:** NS  **Physical:** NS  **Emotional:** NS  **Social:** NS |
| Koutoukidis et al. (2020) | Cancer type: Multiple Myeloma  M^age^: 64.3  M ^Months since diagnosis^: 16.5  Gender: 100% female | I: MASCOT  C: Usual care | I: 38  C: 35 | Individual | 26 | ✓ |  |  | FACT-G | No | Compared mean change.  **Total:** NS  **Physical:** NS  **Emotional:** NS  **Social:** NS |
| Kristensen et al. (2020) | Cancer type: Head and neck  M^age^: 64.3  M ^Months since diagnosis^: N/R  Gender: 35.2% female | I: NUTRI-HAB  C: Waitlist | I: 38  C: 35 | Group | 12 | ✓ | ✓ | ✓ | EORTC QLQ-C30 | No | Compared mean change.  **Total:** NS  **Physical:** NS  **Emotional:** NS  **Social:** NS |
| Kwiatkowski et al. (2017) | Cancer type: Breast  M^age^: 52.0  M ^Months since diagnosis^: N/R  Gender: 100% female | I: SPA  C: Usual care | I: 114  C: 108 | Individual | 2 | ✓ | ✓ | ✓ | SF-16 | Yes | **Total:** I>C  **Physical:** N/R  **Emotional:** N/R  **Social:** N/R |
| Lahart et al. (2016) | Cancer type: Breast  M^age^: 53.6  M ^Months since diagnosis^: 9.5  Gender: 100% female | I: Home based Physical Activity intervention  C: Usual Care | I: 37  C: 33 | Group and telephone | 26 | ✓ |  |  | FACT-G | No | **Total:** NS  **Physical:** NS  **Emotional:** NS  **Social:** NS |
| Ligibel et al. (2012) | Cancer type: Breast, colorectal, and rectal  M^age^: 54.3  M ^Months since diagnosis^: N/R  Gender: 92.6% female | I: AACT  C: Usual Care | I: 48  C: 57 | Telephone | 16 | ✓ |  |  | EORTC QLQC30 | No | Compared mean change.  **Total:** NS  **Physical:** N/R  **Emotional:** N/R  **Social:** N/R |
| Littman et al. (2012) | Cancer type: Breast  M^age^: 56.4  M ^Months since diagnosis^: N/R  Gender: 100% female | I: Yoga  C: Waitlist | I: 30  C: 28 | Group | 24 | ✓ |  |  | FACT-G | Yes | **Total:** NS  **Physical:** NS  **Emotional:** NS  **Social:** NS |
| Livingston et al. (2015) | Cancer type: Prostate  M^age^: 66  M ^Months since diagnosis^: N/R  Gender: 100% male | I: ENGAGE  C: Usual Care | I: 46  C: 83 | Individual | 12 | ✓ |  |  | EORTC QLQC30 | No | **Total:** NS  **Physical:** NS  **Emotional:** NS  **Social:** NS |
| Long Parma et al. (2022) | Cancer type: Breast  M^age^: 55  M ^Months since diagnosis^: 10.7  Gender: 100% female | I: Intervention  C: Usual Care | I: 79  C: 80 | Telephone | 52 |  | ✓ |  | PROMIS QoL | No | **Total:** NS  **Physical:** NS  **Emotional:** NS  **Social:** NS |
| Mardani et al. (2021) | Cancer type: Prostate  M^age^: 69.9  M ^Months since diagnosis^: 10.7  Gender: 100% male | I: Intervention  C: Usual Care | I: 35  C: 36 | Print | 12 | ✓ |  |  | EORTC QLQC30 | Yes | **Total:** NS  **Physical:** I>C  **Emotional:** NS  **Social:** NS |
| McCarroll et al. (2014) | Cancer type: Endometrial  M^age^: 57.9  M ^Months since diagnosis^: 25.6  Gender: 100% female | I: SUCCEED  C: Usual Care | I: 35  C: 36 | Group | 26 | ✓ | ✓ |  | FACT-G | Yes | Compared mean change.  **Total:** NS  **Physical:** I>C  **Emotional:** NS  **Social:** NS |
| McGowan et al. (2013) | Cancer type: Prostate  M^age^: 68.4  M ^Months since diagnosis^: 29.0  Gender: 100% male | I^1^: PROMOTE - self administered  I^2^: PROMOTE - telephone  C: Usual care | I^1^:102  I^2^:103  C:98 | Print  Print and telephone | 12 | ✓  ✓ |  |  | SF-36 | No | **Total:** N/A  **Physical:** NS  **Mental:** NS  **Social:** NS |
| McKenzie et al. (2003) | Cancer type: Breast  M^age^: 56.6  M ^Months since diagnosis^: N/R  Gender: 100% female | I: Intervention  C: Waitlist | I: 7  C: 7 | Individual | 8 | ✓ | ✓ |  | SF-36 | No | **Total:** N/A  **Physical:** NS  **Emotional:** NS  **Social:** NS |
| McNeil et al. (2019) | Cancer type: Breast  M^age^: 58.7  M ^Months since diagnosis^: N/R  Gender: 100% female | I^1^: BC-PAL Lower intensity  I^2^: BC-PAL Higher Intensity  C: Usual Care | I^1^:15  I^2^:15  C:13 | Wearable activity tracker | 12 | ✓ |  |  | FACT-B | No | **Total:** NS  **Physical:** NS  **Emotional:** NS  **Social:** NS |
| Moraes et al. (2021) | Cancer type: Breast  M^age^: 54.6  M ^Months since diagnosis^: 41.7  Gender: 100% female | I: Resistance Training  C: Waitlist | I: 12  C: 13 | Individual | 8 | ✓ |  |  | SF-36 | No | **Total:** NS  **Physical:** I>C  **Emotional:** NS  **Social:** NS |
| Morey et al. (2009) | Cancer type: Breast, prostate, and colorectal  M^age^: 73.1  M ^Months since diagnosis^: 8.6  Gender: 100% female | I: RENEW  C: Waitlist | I: 269  C: 289 | Print, Telephone, SMS | 52 | ✓ | ✓ |  | SF-36 | Yes | **Total:** N/A  **Physical:** NS  **Emotional:** NS  **Social:** NS |
| Mulero Portela et al. (2008) | Cancer type: Breast  M^age^: 52.9  M ^Months since diagnosis^: N/R  Gender: 100% female | I^1^: Gym exercise  I^2^: Home exercise  C: Usual Care | I^1^:12  I^2^:13  C:9 | Individual | 26 | ✓ |  |  | FACT-B | No | **Total:** NS  **Physical:** N/R  **Emotional:** N/R  **Social:** N/R |
| Murtezani et al. (2014) | Cancer type: Breast  M^age^: 52.0  M ^Months since diagnosis^: N/R  Gender: 100% female | I: Exercise group  C: Waitlist | I: 30  C: 32 | Group | 10 | ✓ |  |  | FACT-G | Yes | **Total:** I>C  **Physical:** I>C (NS when comparing change over time)  **Emotional:** NS (I>C when comparing change over time)  **Social:** I>C (NS when comparing change over time) |
| Naumann, Munro, et al. (2012) | Cancer type: Breast  M^age^: 53.6  M ^Months since diagnosis^: 8.0  Gender: 100% female | I^1^: Group Exercise Counselling  I^2^: Individual Exercise Counselling  C: Usual Care | I^1^:14  I^2^:12  C:10 | Group  Individual | 9 | ✓  ✓ |  | ✓  ✓ | FACT-B | Yes | Compared mean change.  **Total:** I^2^>C  **Physical:** I^2^>C  **Emotional:** I^1^>C, I^2^>C  **Social:** NS |
| Naumann, Martin, et al. (2012) | Cancer type: Breast  M^age^: 49.9  M ^Months since diagnosis^: 7.3  Gender: 100% female | I^1^: Exercise  I^2^: Exercise and Counselling  C: Usual Care | I^1^:14  I^2^:12  C:10 | Individual  Individual | 8 | ✓  ✓ |  | ✓ | EORTC QLQ-BR23 | No | **Total:** NS  **Physical:** NS  **Emotional:** I^2^>C  **Social:** NS |
| Ohira et al. (2006) | Cancer type: Breast  M^age^: 53.0  M ^Months since diagnosis^: 22.5  Gender: 100% female | I: Weight Training for Breast Cancer Survivors  C: Waitlist | I: 39  C: 40 | Group | 26 | ✓ |  |  | CARES-SF | Yes | **Total:** NS  **Physical:** C>I  **Emotional:** N/A  **Social:** N/A |
| O'Neill et al. (2018) | Cancer type: Esophageal, esophagogastric junction, or gastric  M^age^: 65.6  M ^Months since diagnosis^: N/R  Gender: 37.8% female | I: RESTORE  C: Usual care | I: 20  C: 19 | Group | 12 | ✓ | ✓ | ✓ | EORTC QLQ-C30 | No | **Total:** NS  **Physical:** NS  **Emotional:** NS  **Social:** NS |
| Park et al. (2015) | Cancer type: Breast and Colorectal  M^age^: 51.8  M ^Months since diagnosis^: 23.1  Gender: 88.3% female | I: Oncologist’s Exercise Recommendation With Exercise Motivation Package  C: Waitlist | I: 50  C: 59 | Group, DVD, pedometer | 4 | ✓ |  |  | EORTC QLQ-C30 | No | Compared mean change.  **Total:** NS  **Physical:** NS  **Emotional:** NS  **Social:** NS |
| Park et al. (2019) | Cancer type: Breast, Gynecological, lymphoma, colorectal  M^age^: 51.9  M ^Months since diagnosis^: N/R  Gender: 37.8% female | I: FIT  C: Usual Care | I: 62  C: 64 | Individual | 24 | ✓ |  |  | FACT-G | No | Divided groups by participants taking aromatase inhibitors, tamoxifen, and no endocrine therapy.  **Total:** NS  **Physical:** NS  **Emotional:** I>C only for those not taking endocrine therapy  **Social:** NS |
| Pisu et al. (2017) | Cancer type: Endometrial, ovarian, breast, colorectal  M^age^: 57.9  M ^Months since diagnosis^: 50.7  Gender: 100% female | I: Rhythm  C: Waitlist | I: 62  C: 64 | Individual | 12 | ✓ |  |  | SF-36 | No | **Total:** N/A  **Physical:** I>C  **Emotional:** NS  **Social:** NS |
| Prinsen et al. (2013) | Cancer type: Breast, head and neck, non-hodgkin, prostate, testicular, and thyroid  M^age^: 49.3  M ^Months since diagnosis^: 49.6  Gender: 100% female | I: CBT  C: Waitlist | I: 23  C: 14 | Individual | 24 | ✓ |  | ✓ | Sickness Impact Profile 8 | No | **Total:** I>C  **Physical:** N/A  **Emotional:** N/A  **Social:** N/A |
| Reeves et al. (2017) | Cancer type: Breast  M^age^: 55.3  M ^Months since diagnosis^: 15.9  Gender: 100% female | I: Living well after Breast Cancer  C: Usual care | I: 40  C: 34 | Telephone | 26 | ✓ | ✓ |  | SF-36 | No | **Total:** N/A  **Physical:** N/R  **Emotional:** N/R  **Social:** N/R  **Other:**  Physical Composite: NS  Emotional composite: NS |
| Reeves et al. (2021) | Cancer type: Breast  M^age^: 55.0  M ^Months since diagnosis^: 10.7  Gender: 100% female | I: Lifestyle Intervention  C: Usual care | I: 79  C: 80 | Telephone | 52 | ✓ | ✓ |  | PROMIS QoL | No | **Total:** N/A  **Physical:** N/R  **Emotional:** N/R  **Social:** N/R  **Other:**  Physical Composite: NS  Mental composite: NS |
| Rogers et al. (2009) | Cancer type: Breast  M^age^: 53.0  M ^Months since diagnosis^: N/R  Gender: 100% female | I: BEAT  C: Usual care | I: 20  C: 19 | Individual | 12 | ✓ |  |  | FACT-G | No | **Total:** NS  **Physical:** NS  **Emotional: NS**  **Social:** NS (I>C when comparing change over time) |
| Rogers et al. (2015) | Cancer type: Breast  M^age^: 54.4  M ^Months since diagnosis^: N/R  Gender: 100% female | I: BEAT  C: Usual care | I: 105  C: 108 | Individual | 12 | ✓ |  | ✓ | FACT-G | No | **Total:** I>C  **Physical:** I>C  **Emotional:** I>C  **Social:** NS |
| Ruiz-Vozmediano et al. (2020) | Cancer type: Breast  M^age^: 50.1  M ^Months since diagnosis^: N/R  Gender: 100% female | I: Intervention  C: Usual Care | I: 31  C: 32 | Group | 26 | ✓ | ✓ | ✓ | EORTC QLQ-C30 | Yes | **Total:** NS  **Physical:** NS (I>C when comparing change over time)  **Emotional:** N/R  **Social:** NS |
| Saarto et al. (2012) | Cancer type: Breast  M^age^: 52.4  M ^Months since diagnosis^: N/R  Gender: 100% female | I: Intervention  C: Usual Care | I: 263  C: 237 | Group | 52 | ✓ |  |  | EORTC QLQ-C30 | Yes | Compared mean change.  **Total:** NS  **Physical:** NS  **Emotional:** NS  **Social:** C>I |
| Sandel et al. (2005) | Cancer type: Breast  M^age^: 59.6  M ^Months since diagnosis^: N/R  Gender: 100% female | I: Intervention  C: Usual Care | I: 19  C: 16 | Group | 12 | ✓ |  |  |  | Yes | **Total:** NS (I>C when comparing groups over time)  **Physical:** N/R  **Emotional:** N/R  **Social:** N/R |
| Scott et al. (2013) | Cancer type: Breast  M^age^: 55.7  M ^Months since diagnosis^: N/R  Gender: 100% female | I: Pragmatic lifestyle intervention  C: Usual Care | I: 47  C: 43 | Individual and print | 26 | ✓ | ✓ |  | FACT-B | No | **Total:** NS  **Physical:** N/R  **Emotional:** N/R  **Social:** N/R |
| Shobeiri et al. (2016) | Cancer type: Breast  M^age^: 43.1  M ^Months since diagnosis^: NR  Gender: 100% female | I: Lifestyle Intervention  C: Usual care | I: 26  C: 27 | Group | 10 | ✓ |  |  | EORTC QLQ-C30 | Yes | **Total:** I>C  **Physical:** I>C  **Emotional:** I>C  **Social:** NS |
| Short et al. (2015) | Cancer type: Breast  M^age^: 55.0  M ^Months since diagnosis^: N/R  Gender: 100% female | I^1^: Move more for life tailored  I^2^: Move more for life targeted  C: Usual Care | I^1^:91  I^2^:92  C:93 | Print | 12 | ✓  ✓ |  |  | FACT-G | No | **Total:** NS  **Physical:** NS  **Emotional:** NS  **Social:** NS |
| Singleton et al. (2022) | Cancer type: Breast  M^age^: 55.1  M ^Months since diagnosis^: N/R  Gender: 100% female | I: EMPOWER-SMS  C: Usual care | I: 64  C: 71 | SMS | 26 | ✓ | ✓ | ✓ | EORTC QLQ-C30 | No | **Total:** NS  **Physical:** I>C  **Emotional:** NS  **Social:** NS |
| Speck et al. (2010) | Cancer type: Breast  M^age^: 56.5  M ^Months since diagnosis^: 60.7  Gender: 100% female | I: EMPOWER-SMS  C: Usual care | I: 64  C: 71 | Individual | 52 | ✓ |  |  | SF-36 | No | **Total:** N/A  **Physical:** N/R  **Emotional:** N/R  **Social:** N/R  **Other:**  Physical Composite: NS  Mental composite: NS for overall sample, however, I>C for those with lymphedema |
| Strunk et al. (2018) | Cancer type: Breast  M^age^: 53.1  M ^Months since diagnosis^: 44.8  Gender: 100% female | I: Intervention  C: Waitlist | I: 26  C: 25 | Group | 52 | ✓ |  |  | EORTC QLQ-C30 | Yes | **Total:** NS  **Physical:** NS  **Emotional:** NS  **Social:** NS |
| Swisher et al. (2015) | Cancer type: Breast  M^age^: 53.7  M ^Months since diagnosis^: 44.8  Gender: 100% female | I: Get Fit for the Fight  C: Usual Care | I: 13  C: 10 | Individual | 12 | ✓ | ✓ |  | FACT-B | No | **Total:** NS  **Physical:** NS  **Emotional:** NS  **Social:** NS |
| Thorsen et al. (2005) | Cancer type: Lymphomas, breast, gynecologic, or testicular  M^age^: 39.1  M ^Months since diagnosis^: N/R  Gender: 68% female | I: Get Fit for the Fight  C: Usual Care | I: 59  C: 52 | Individual and print | 14 | ✓ |  |  | EORTC QLQ-C30 | No | Compared mean change  **Total:** NS  **Physical:** NS  **Emotional:** NS  **Social:** NS |
| Toohey et al. (2018) | Cancer type: Breast, Ovarian, Appendix, Anal, Cervical, Liver, Oesophageal, Melanoma, Leiomyosarcoma  M^age^: 51.5  M ^Months since diagnosis^: N/R  Gender: 88% female | I^1^: Low volume high-intensity interval training  I^2^: Continuous low to moderate-intensity training  C: Usual Care | I^1^:24  I^2^:21  C:12 | Individual | 12 | ✓  ✓ |  |  | FACT-G | Yes | Compared within group effect sizes  **Total:** I^1^>C, I^1^> I^2^  **Physical:** I^1^>C, I^2^>C  **Emotional:** I^1^>C, I^1^> I^2^  **Social:** NS |
| Vallance et al. (2008) | Cancer type: Breast  M^age^: 58.0  M^Months since diagnosis^: 39.0  Gender: 100% female | I^1^: Phyical Activity print  I^2^: Physical activity pedometer  I^3^: Physical activity combination  C: Usual Care | I^1^: 62  I^2^: 69  I^3^: 67  C: 68 | Print  Pedometer  Combination |  | ✓  ✓  ✓ |  |  | FACT-B | No | **Total:** NS  **Physical:** NS  **Emotional:** NS  **Social:** NS |
| Vallance et al. (2020) | Cancer type: Breast  M^age^: 62.0  M ^Months since diagnosis^: N/R  Gender: 100% female | I: Get Fit for the Fight  C: Waitlist | I: 40  C: 40 | Face to face, wearable tracker, telephone | 12 | ✓ |  |  | FACT-G | Yes | **Total:** NS  **Physical:** N/R  **Emotional:** N/R  **Social:** N/R |
| Vallerand et al. (2018) | Cancer type: Leukemia, Hodgkin Lymphoma, Non-Hodgkin Lymphoma  M^age^: 56.2  M ^Months since diagnosis^: 87.6  Gender: 61% female | I: telephone counselling exercise  C: Usual Care | I: 26  C: 25 | Telephone | 12 | ✓ |  | ✓ | SF-36 | No | **Total:** N/A  **Physical:** NS  **Emotional:** NS  **Social:** NS |
| van de Wiel et al. (2021) | Cancer type: Leukemia, Hodgkin Lymphoma, Non-Hodgkin Lymphoma  M^age^: 56.2  M ^Months since diagnosis^: 87.6  Gender: 61% female | I^1^: Internet-based PA Support program (IPAS)  I^2^: IPAS + support  C: Usual Care | I^1^:24  I^2^:28  C:32 | Online  Online and telephone | 26 | ✓  ✓ |  |  | SF-36 | No | **Total:** N/A  **Physical:** NS  **Emotional:** NS (I>C when both interventions combined into one group)  **Social:** NS |
| von Gruenigen et al. (2009) | Cancer type: Breast and prostate  M^age^: 54.73  M ^Months since diagnosis^: 23.65  Gender: 48.9% female | I: Lifestyle Intervention  C: Usual Care | I: 23  C: 22 | Group | 26 | ✓ | ✓ | ✓ | FACT-G | No | **Total:** NS  **Physical:** NS  **Emotional:** NS  **Social:** NS |
| Wang et al. (2021) | Cancer type: Breast  M^age^: 55.8  M ^Months since diagnosis^: 22.9  Gender: 100% female | I: Lifestyle Intervention  C: Waitlist | I: 23  C: 22 | Individual and DVD | 18 | ✓ |  |  | FACT-ES | No | Compared mean change.  **Total:** NS  **Physical:** I>C  **Emotional:** NS  **Social:** NS |
| Willems et al. (2017) | Cancer type: All  M^age^: 56.5  M ^Months since diagnosis^: NR  Gender: 81% female | I: Kanker Nazorg Wijzer  C: Waitlist | I: 188  C: 121 | Online | 26 | ✓ | ✓ | ✓ | EORTC QLQ-C30 | Yes | **Total:** NS  **Physical:** NS  **Emotional:** I>C (NS when missing data accounted for)  **Social:** I>C (NS when missing data accounted for) |
| Winkels et al. (2017) | Cancer type: Breast  M^age^: 59.4  M^Months since diagnosis^: 91.9  Gender: 100% female | I^1^: Exercise  I^2^: Weightloss  I^3^: exercise + weightloss  C: Usual Care | I^1^: 62  I^2^: 69  I^3^: 67  C: 68 | Group | 52 | ✓  ✓ | ✓  ✓ |  | SF-36 | No | **Total:** N/A  **Physical:** NS  **Emotional:** NS  **Social:** NS |
| Winters-Stone et al. (2016) | Cancer type: Prostate  M^age^: 56.5  M ^Months since diagnosis^: 6.4  Gender: 100% male | I: Kanker Nazorg Wijzer  C: Waitlist | I: 32  C: 32 | Individual | 26 | ✓ |  |  | SF-36 | No | **Total:** N/A  **Physical:** I>C  **Emotional:** NS  **Social:** N/R |

**References**

Adams, S. C., DeLorey, D. S., Davenport, M. H., Fairey, A. S., North, S., & Courneya, K. S. (2018). Effects of high-intensity interval training on fatigue and quality of life in testicular cancer survivors. *British journal of cancer, 118*(10), 1313-1321. <https://doi.org/https://dx.doi.org/10.1038/s41416-018-0044-7>

Alibhai, S. M. H., O'Neill, S., Fisher-Schlombs, K., Breunis, H., Timilshina, N., Brandwein, J. M., Minden, M. D., Tomlinson, G. A., & Culos-Reed, S. N. (2014). A pilot phase II RCT of a home-based exercise intervention for survivors of AML. *Supportive care in cancer : official journal of the Multinational Association of Supportive Care in Cancer, 22*(4), 881-889. <https://doi.org/https://dx.doi.org/10.1007/s00520-013-2044-8>

Bail, J. R., Fruge, A. D., Cases, M. G., De Los Santos, J. F., Locher, J. L., Smith, K. P., Cantor, A. B., Cohen, H. J., & Demark-Wahnefried, W. (2018). A home-based mentored vegetable gardening intervention demonstrates feasibility and improvements in physical activity and performance among breast cancer survivors. *Cancer, 124*(16), 3427-3435. <https://doi.org/https://dx.doi.org/10.1002/cncr.31559>

Baruth, M., Wilcox, S., Der Ananian, C., & Heiney, S. (2015). Effects of Home-Based Walking on Quality of Life and Fatigue Outcomes in Early Stage Breast Cancer Survivors: A 12-Week Pilot Study. *Journal of physical activity & health, 12 Suppl 1*(101189457), S110-118. <https://doi.org/https://dx.doi.org/10.1123/jpah.2012-0339>

Basen-Engquist, K., Taylor, C. L. C., Rosenblum, C., Smith, M. A., Shinn, E. H., Greisinger, A., Gregg, X., Massey, P., Valero, V., & Rivera, E. (2006). Randomized pilot test of a lifestyle physical activity intervention for breast cancer survivors. *Patient education and counseling, 64*(1-3), 225-234. <https://www.sciencedirect.com/science/article/abs/pii/S0738399106000498?via%3Dihub>

<https://www.sciencedirect.com/science/article/pii/S0738399106000498?via%3Dihub>

Blair, C. K., Harding, E., Wiggins, C., Kang, H., Schwartz, M., Tarnower, A., Du, R., & Kinney, A. Y. (2021). A home-based mobile health intervention to replace sedentary time with light physical activity in older cancer survivors: Randomized controlled pilot trial. *JMIR Cancer, 7*(2). <https://doi.org/10.2196/18819>

Bourke, L., Thompson, G., Gibson, D. J., Daley, A., Crank, H., Adam, I., Shorthouse, A., & Saxton, J. (2011). Pragmatic Lifestyle Intervention in Patients Recovering From Colon Cancer: A Randomized Controlled Pilot Study. *Archives of Physical Medicine & Rehabilitation, 92*(5), 749-755. <https://doi.org/10.1016/j.apmr.2010.12.020>

Braakhuis, A., Campion, P., & Bishop, K. (2017). The Effects of Dietary Nutrition Education on Weight and Health Biomarkers in Breast Cancer Survivors. *Medical sciences (Basel, Switzerland), 5*(2). <https://doi.org/https://dx.doi.org/10.3390/medsci5020012>

Broderick, J. M., Guinan, E., Kennedy, M. J., Hollywood, D., Courneya, K. S., Culos-Reed, S. N., Bennett, K., O' Donnell, D. M., & Hussey, J. (2013). Feasibility and efficacy of a supervised exercise intervention in de-conditioned cancer survivors during the early survivorship phase: the PEACH trial. *Journal of cancer survivorship : research and practice, 7*(4), 551-562. <https://doi.org/https://dx.doi.org/10.1007/s11764-013-0294-6>

Brown, J. C., Damjanov, N., Courneya, K. S., Troxel, A. B., Zemel, B. S., Rickels, M. R., Ky, B., Rhim, A. D., Rustgi, A. K., & Schmitz, K. H. (2018). A randomized dose-response trial of aerobic exercise and health-related quality of life in colon cancer survivors. *Psycho-Oncology, 27*(4), 1221-1228. <https://doi.org/https://dx.doi.org/10.1002/pon.4655>

Brown, J. C., Giobbie-Hurder, A., Yung, R. L., Mayer, E. L., Tolaney, S. M., Partridge, A. H., & Ligibel, J. A. (2022). The effects of a clinic-based weight loss program on health-related quality of life and weight maintenance in cancer survivors: A randomized controlled trial. *Psycho-Oncology, 31*(2), 326-333. <https://doi.org/https://dx.doi.org/10.1002/pon.5817>

Brown, J. C., Sarwer, D. B., Troxel, A. B., Sturgeon, K., DeMichele, A. M., Denlinger, C. S., & Schmitz, K. H. (2021). A randomized trial of exercise and diet on health-related quality of life in survivors of breast cancer with overweight or obesity. *Cancer*. <https://doi.org/10.1002/cncr.33752>

Burnham, T. R., & Wilcox, A. (2002). Effects of exercise on physiological and psychological variables in cancer survivors. *Medicine and science in sports and exercise, 34*(12), 1863-1867.

Casla, S., López-Tarruella, S., Jerez, Y., Marquez-Rodas, I., Galvao, D. A., Newton, R. U., Cubedo, R., Calvo, I., Sampedro, J., & Barakat, R. (2015). Supervised physical exercise improves VO 2max, quality of life, and health in early stage breast cancer patients: a randomized controlled trial. *Breast cancer research and treatment, 153*(2), 371-382. <https://link.springer.com/article/10.1007%2Fs10549-015-3541-x>

<https://link.springer.com/content/pdf/10.1007/s10549-015-3541-x.pdf>

Chang, Y.-L., Tsai, Y.-F., Hsu, C.-L., Chao, Y.-K., Hsu, C.-C., & Lin, K.-C. (2020). The effectiveness of a nurse-led exercise and health education informatics program on exercise capacity and quality of life among cancer survivors after esophagectomy: A randomized controlled trial. *International journal of nursing studies, 101*(gs8, 0400675), 103418. <https://doi.org/https://dx.doi.org/10.1016/j.ijnurstu.2019.103418>

Courneya, K. S., Mackey, J. R., Bell, G. J., Jones, L. W., Field, C. J., & Fairey, A. S. (2003). Randomized controlled trial of exercise training in postmenopausal breast cancer survivors: cardiopulmonary and quality of life outcomes. *Journal of clinical oncology : official journal of the American Society of Clinical Oncology, 21*(9), 1660-1668.

Cramer, H., Rabsilber, S., Lauche, R., Kummel, S., & Dobos, G. (2015). Yoga and meditation for menopausal symptoms in breast cancer survivors-A randomized controlled trial. *Cancer, 121*(13), 2175-2184. <https://doi.org/http://dx.doi.org/10.1002/cncr.29330http://dx.doi.org/10.1002/cncr.29330>

Cuesta-Vargas, A. I., Buchan, J., & Arroyo-Morales, M. (2014). A multimodal physiotherapy programme plus deep water running for improving cancer-related fatigue and quality of life in breast cancer survivors. *European journal of cancer care, 23*(1), 15-21. <https://doi.org/https://dx.doi.org/10.1111/ecc.12114>

Culos-Reed, S. N., Carlson, L. E., Daroux, L. M., & Hately-Aldous, S. (2006). A pilot study of yoga for breast cancer survivors: Physical and psychological benefits. *Psycho-Oncology, 15*(10), 891-897. <https://doi.org/http://dx.doi.org/10.1002/pon.1021http://dx.doi.org/10.1002/pon.1021>

Culos-Reed, S. N., Robinson, J. W., Lau, H., Stephenson, L., Keats, M., Norris, S., Kline, G., Faris, P., Culos-Reed, S. N., Robinson, J. W., Lau, H., Stephenson, L., Keats, M., Norris, S., Kline, G., & Faris, P. (2010). Physical activity for men receiving androgen deprivation therapy for prostate cancer: benefits from a 16-week intervention. *Supportive Care in Cancer, 18*(5), 591-599. <https://doi.org/10.1007/s00520-009-0694-3>

Daley, A. J., Crank, H., Saxton, J. M., Mutrie, N., Coleman, R., & Roalfe, A. (2007). Randomized trial of exercise therapy in women treated for breast cancer. *Journal of Clinical Oncology, 25*(13), 1713-1721.

De Luca, V., Minganti, C., Borrione, P., Grazioli, E., Cerulli, C., Guerra, E., Bonifacino, A., & Parisi, A. (2016). Effects of concurrent aerobic and strength training on breast cancer survivors: a pilot study. *Public health, 136*(qi7, 0376507), 126-132. <https://doi.org/https://dx.doi.org/10.1016/j.puhe.2016.03.028>

Demark-Wahnefried, W., Cases, M. G., Cantor, A. B., Fruge, A. D., Smith, K. P., Locher, J., Cohen, H. J., Tsuruta, Y., Daniel, M., Kala, R., & De Los Santos, J. F. (2018). Pilot Randomized Controlled Trial of a Home Vegetable Gardening Intervention among Older Cancer Survivors Shows Feasibility, Satisfaction, and Promise in Improving Vegetable and Fruit Consumption, Reassurance of Worth, and the Trajectory of Central Adipos. *Journal of the Academy of Nutrition and Dietetics, 118*(4), 689-704. <https://doi.org/https://dx.doi.org/10.1016/j.jand.2017.11.001>

Dieli-Conwright, C. M., Courneya, K. S., Demark-Wahnefried, W., Sami, N., Lee, K., Sweeney, F. C., Stewart, C., Buchanan, T. A., Spicer, D., Tripathy, D., Bernstein, L., & Mortimer, J. E. (2018). Aerobic and resistance exercise improves physical fitness, bone health, and quality of life in overweight and obese breast cancer survivors: a randomized controlled trial. *Breast cancer research : BCR, 20*(1), 124. <https://doi.org/https://dx.doi.org/10.1186/s13058-018-1051-6>

Fillion, L., Gagnon, P., Leblond, F., Gelinas, C., Savard, J., Dupuis, R., Duval, K., & Larochelle, M. (2008). A brief intervention for fatigue management in breast cancer survivors. *Cancer Nursing, 31*(2), 145-159. <https://doi.org/http://dx.doi.org/10.1097/01.NCC.0000305698.97625.95http://dx.doi.org/10.1097/01.NCC.0000305698.97625.95>

Galiano-Castillo, N., Cantarero-Villanueva, I., Fernandez-Lao, C., Ariza-Garcia, A., Diaz-Rodriguez, L., Del-Moral-Avila, R., & Arroyo-Morales, M. (2016). Telehealth system: A randomized controlled trial evaluating the impact of an internet-based exercise intervention on quality of life, pain, muscle strength, and fatigue in breast cancer survivors. *Cancer, 122*(20), 3166-3174. <https://doi.org/https://dx.doi.org/10.1002/cncr.30172>

Garcia-Soidan, J. L., Perez-Ribao, I., Leiros-Rodriguez, R., & Soto-Rodriguez, A. (2020). Long-Term Influence of the Practice of Physical Activity on the Self-Perceived Quality of Life of Women with Breast Cancer: A Randomized Controlled Trial. *International journal of environmental research and public health, 17*(14). <https://doi.org/https://dx.doi.org/10.3390/ijerph17144986>

Ghavami, H., & Akyolcu, N. (2017). Effects of a lifestyle interventions program on quality of life in breast cancer survivors. *UHOD - Uluslararasi Hematoloji-Onkoloji Dergisi, 27*(2), 91-99. <https://doi.org/10.4999/uhod.171734>

Golsteijn, R. H. J., Bolman, C., Volders, E., Peels, D. A., de Vries, H., & Lechner, L. (2018). Short-term efficacy of a computer-tailored physical activity intervention for prostate and colorectal cancer patients and survivors: a randomized controlled trial. *The international journal of behavioral nutrition and physical activity, 15*(1), 106. <https://doi.org/https://dx.doi.org/10.1186/s12966-018-0734-9>

Gorzelitz, J. S., Stoller, S., Costanzo, E., Gangnon, R., Koltyn, K., Dietz, A. T., Spencer, R. J., Rash, J., & Cadmus-Bertram, L. (2022). Improvements in strength and agility measures of functional fitness following a telehealth-delivered home-based exercise intervention in endometrial cancer survivors. *Supportive care in cancer : official journal of the Multinational Association of Supportive Care in Cancer, 30*(1), 447-455. <https://doi.org/https://dx.doi.org/10.1007/s00520-021-06415-2>

Hagstrom, A. D., Marshall, P. W. M., Lonsdale, C., Cheema, B. S., Fiatarone Singh, M. A., & Green, S. (2016). Resistance training improves fatigue and quality of life in previously sedentary breast cancer survivors: a randomised controlled trial. *European journal of cancer care, 25*(5), 784-794. <https://doi.org/https://dx.doi.org/10.1111/ecc.12422>

Herrero, F., San Juan, A. F., Fleck, S. J., Balmer, J., Perez, M., Canete, S., Earnest, C. P., Foster, C., & Lucia, A. (2006). Combined aerobic and resistance training in breast cancer survivors: A randomized, controlled pilot trial. *International journal of sports medicine, 27*(7), 573-580. <https://www.thieme-connect.com/products/ejournals/abstract/10.1055/s-2005-865848>

<https://www.thieme-connect.com/products/ejournals/pdf/10.1055/s-2005-865848.pdf>

Ho, M., Ho, J. W. C., Fong, D. Y. T., Lee, C. F., Macfarlane, D. J., Cerin, E., Lee, A. M., Leung, S., Chan, W. Y. Y., Leung, I. P. F., Lam, S. H. S., Chu, N., Taylor, A. J., & Cheng, K.-K. (2020). Effects of dietary and physical activity interventions on generic and cancer-specific health-related quality of life, anxiety, and depression in colorectal cancer survivors: a randomized controlled trial. *Journal of cancer survivorship : research and practice, 14*(4), 424-433. <https://doi.org/https://dx.doi.org/10.1007/s11764-020-00864-0>

Holtdirk, F., Mehnert, A., Weiss, M., Mayer, J., Meyer, B., Brode, P., Claus, M., & Watzl, C. (2021). Results of the Optimune trial: A randomized controlled trial evaluating a novel Internet intervention for breast cancer survivors. *PloS one, 16*(5), e0251276. <https://doi.org/https://dx.doi.org/10.1371/journal.pone.0251276>

Kampshoff, C. S., Chinapaw, M. J. M., Brug, J., Twisk, J. W. R., Schep, G., Nijziel, M. R., van Mechelen, W., & Buffart, L. M. (2015). Randomized controlled trial of the effects of high intensity and low-to-moderate intensity exercise on physical fitness and fatigue in cancer survivors: results of the Resistance and Endurance exercise After ChemoTherapy (REACT) study. *BMC medicine, 13*(101190723), 275. <https://doi.org/https://dx.doi.org/10.1186/s12916-015-0513-2>

Kim, J. Y., Lee, M. K., Lee, D. H., Kang, D. W., Min, J. H., Lee, J. W., Chu, S. H., Cho, M. S., Kim, N. K., & Jeon, J. Y. (2019). Effects of a 12-week home-based exercise program on quality of life, psychological health, and the level of physical activity in colorectal cancer survivors: a randomized controlled trial. *Supportive care in cancer : official journal of the Multinational Association of Supportive Care in Cancer, 27*(8), 2933-2940. <https://doi.org/https://dx.doi.org/10.1007/s00520-018-4588-0>

Kim, S. H., Shin, M. S., Lee, H. S., Lee, E. S., Ro, J. S., Kang, H. S., Kim, S. W., Lee, W. H., Kim, H. S., Kim, C. J., Kim, J., & Yun, Y. H. (2011). Randomized pilot test of a simultaneous stage-matched exercise and diet intervention for breast cancer survivors. *Oncology nursing forum, 38*(2), E97-106. <https://doi.org/https://dx.doi.org/10.1188/11.ONF.E97-E106>

Koutoukidis, D. A., Beeken, R. J., Manchanda, R., Burnell, M., Ziauddeen, N., Michalopoulou, M., Knobf, M. T., & Lanceley, A. (2019). Diet, physical activity, and health-related outcomes of endometrial cancer survivors in a behavioral lifestyle program: the Diet and Exercise in Uterine Cancer Survivors (DEUS) parallel randomized controlled pilot trial. *International journal of gynecological cancer : official journal of the International Gynecological Cancer Society, 29*(3), 531-540. <https://doi.org/https://dx.doi.org/10.1136/ijgc-2018-000039>

Koutoukidis, D. A., Land, J., Hackshaw, A., Heinrich, M., McCourt, O., Beeken, R. J., Philpott, S., DeSilva, D., Rismani, A., Rabin, N., Popat, R., Kyriakou, C., Papanikolaou, X., Mehta, A., Paton, B., Fisher, A., & Yong, K. L. (2020). Fatigue, quality of life and physical fitness following an exercise intervention in multiple myeloma survivors (MASCOT): an exploratory randomised Phase 2 trial utilising a modified Zelen design. *British journal of cancer, 123*(2), 187-195. <https://doi.org/https://dx.doi.org/10.1038/s41416-020-0866-y>

Kristensen, M. B., Wessel, I., Beck, A. M., Dieperink, K. B., Mikkelsen, T. B., Moller, J.-J. K., & Zwisler, A.-D. (2020). Effects of a Multidisciplinary Residential Nutritional Rehabilitation Program in Head and Neck Cancer Survivors-Results from the NUTRI-HAB Randomized Controlled Trial. *Nutrients, 12*(7). <https://doi.org/https://dx.doi.org/10.3390/nu12072117>

Kwiatkowski, F., Mouret-Reynier, M.-A., Duclos, M., Bridon, F., Hanh, T., Van Praagh-Doreau, I., Travade, A., Vasson, M.-P., Jouvency, S., Roques, C., & Bignon, Y.-J. (2017). Long-term improvement of breast cancer survivors' quality of life by a 2-week group physical and educational intervention: 5-year update of the 'PACThe' trial. *British journal of cancer, 116*(11), 1389-1393. <https://doi.org/https://dx.doi.org/10.1038/bjc.2017.112>

Lahart, I. M., Metsios, G. S., Nevill, A. M., Kitas, G. D., & Carmichael, A. R. (2016). Randomised controlled trial of a home-based physical activity intervention in breast cancer survivors. *BMC cancer, 16*(100967800), 234. <https://doi.org/https://dx.doi.org/10.1186/s12885-016-2258-5>

Ligibel, J. A., Meyerhardt, J., Pierce, J. P., Najita, J., Shockro, L., Campbell, N., Newman, V. A., Barbier, L., Hacker, E., Wood, M., Marshall, J., Paskett, E., & Shapiro, C. (2012). Impact of a telephone-based physical activity intervention upon exercise behaviors and fitness in cancer survivors enrolled in a cooperative group setting. *Breast cancer research and treatment, 132*(1), 205-213. <https://doi.org/https://dx.doi.org/10.1007/s10549-011-1882-7>

Littman, A. J., Bertram, L. C., Ceballos, R., Ulrich, C. M., Ramaprasad, J., McGregor, B., & McTiernan, A. (2012). Randomized controlled pilot trial of yoga in overweight and obese breast cancer survivors: effects on quality of life and anthropometric measures. *Supportive care in cancer : official journal of the Multinational Association of Supportive Care in Cancer, 20*(2), 267-277. <https://doi.org/https://dx.doi.org/10.1007/s00520-010-1066-8>

Livingston, P. M., Craike, M. J., Salmon, J., Courneya, K. S., Gaskin, C. J., Fraser, S. F., Mohebbi, M., Broadbent, S., Botti, M., Kent, B., & the, E. U.-O. C. G. (2015, 2015/08/01). Effects of a clinician referral and exercise program for men who have completed active treatment for prostate cancer: A multicenter cluster randomized controlled trial (ENGAGE) [<https://doi.org/10.1002/cncr.29385>]. *Cancer, 121*(15), 2646-2654. <https://doi.org/https://doi.org/10.1002/cncr.29385>

Long Parma, D. A., Reynolds, G. L., Munoz, E., & Ramirez, A. G. (2022). Effect of an anti-inflammatory dietary intervention on quality of life among breast cancer survivors. *Supportive care in cancer : official journal of the Multinational Association of Supportive Care in Cancer, 30*(7), 5903-5910. <https://doi.org/https://dx.doi.org/10.1007/s00520-022-07023-4>

Mardani, A., Pedram Razi, S., Mazaheri, R., Haghani, S., & Vaismoradi, M. (2021). Effect of the exercise programme on the quality of life of prostate cancer survivors: A randomized controlled trial. *International Journal of Nursing Practice (John Wiley & Sons, Inc.), 27*(2), 1-11. <https://doi.org/10.1111/ijn.12883>

McCarroll, M. L., Armbruster, S., Frasure, H. E., Gothard, M. D., Gil, K. M., Kavanagh, M. B., Waggoner, S., & von Gruenigen, V. E. (2014). Self-efficacy, quality of life, and weight loss in overweight/obese endometrial cancer survivors (SUCCEED): a randomized controlled trial. *Gynecologic oncology, 132*(2), 397-402. <https://doi.org/https://dx.doi.org/10.1016/j.ygyno.2013.12.023>

McGowan, E. L., North, S., & Courneya, K. S. (2013). Randomized controlled trial of a behavior change intervention to increase physical activity and quality of life in prostate cancer survivors. *Annals of behavioral medicine : a publication of the Society of Behavioral Medicine, 46*(3), 382-393. <https://doi.org/https://dx.doi.org/10.1007/s12160-013-9519-1>

McKenzie, D. C., Kalda, A. L., McKenzie, D. C., & Kalda, A. L. (2003). Effect of upper extremity exercise on secondary lymphedema in breast cancer patients: a pilot study. *Journal of Clinical Oncology, 21*(3), 463-466. <http://ezproxy.flinders.edu.au/login?url=http://search.ebscohost.com/login.aspx?direct=true&db=cin20&AN=106737654&site=ehost-live>

McNeil, J., Brenner, D. R., Stone, C. R., O'Reilly, R., Ruan, Y., Vallance, J. K., Courneya, K. S., Thorpe, K. E., Klein, D. J., & Friedenreich, C. M. (2019). Activity Tracker to Prescribe Various Exercise Intensities in Breast Cancer Survivors. *Medicine & Science in Sports & Exercise, 51*(5), 930-940. <https://doi.org/10.1249/MSS.0000000000001890>

Moraes, R. F., Ferreira-Junior, J. B., Marques, V. A., Vieira, A., Lira, C. A. B., Campos, M. H., Freitas-Junior, R., Rahal, R. M. S., Gentil, P., & Vieiral, C. A. (2021). Resistance Training, Fatigue, Quality of Life, Anxiety in Breast Cancer Survivors. *Journal of Strength & Conditioning Research (Lippincott Williams & Wilkins), 35*(5), 1350-1356. <https://doi.org/10.1519/jsc.0000000000003817>

Morey, M. C., Snyder, D. C., Sloane, R., Cohen, H. J., Peterson, B., Hartman, T. J., Miller, P., Mitchell, D. C., & Demark-Wahnefried, W. (2009). Effects of home-based diet and exercise on functional outcomes among older, overweight long-term cancer survivors: RENEW: a randomized controlled trial. *JAMA, 301*(18), 1883-1891. <https://doi.org/https://dx.doi.org/10.1001/jama.2009.643>

Mulero Portela, A. L., Colón Santaella, C. L., Cruz Gómez, C., & Burch, A. (2008). Feasibility of an exercise program for Puerto Rican women who are breast cancer survivors. *Rehabilitation Oncology, 26*(2), 20-31. <https://doi.org/10.1097/01893697-200826020-00003>

Murtezani, A., Ibraimi, Z., Bakalli, A., Krasniqi, S., Disha, E. D., & Kurtishi, I. (2014). The effect of aerobic exercise on quality of life among breast cancer survivors: a randomized controlled trial. *Journal of cancer research and therapeutics, 10*(3), 658-664. <https://doi.org/https://dx.doi.org/10.4103/0973-1482.137985>

Naumann, F., Martin, E., Philpott, M., Smith, C., Groff, D., & Battaglini, C. (2012). Can counseling add value to an exercise intervention for improving quality of life in breast cancer survivors? A feasibility study. *The journal of supportive oncology, 10*(5), 188-194. <https://doi.org/https://dx.doi.org/10.1016/j.suponc.2011.09.004>

Naumann, F., Munro, A., Martin, E., Magrani, P., Buchan, J., Smith, C., Piggott, B., & Philpott, M. (2012). An individual-based versus group-based exercise and counselling intervention for improving quality of life in breast cancer survivors. A feasibility and efficacy study. *Psycho-Oncology, 21*(10), 1136-1139. <https://doi.org/https://dx.doi.org/10.1002/pon.2015>

O'Neill, L. M., Guinan, E., Doyle, S. L., Bennett, A. E., Murphy, C., Elliott, J. A., O'Sullivan, J., Reynolds, J. V., & Hussey, J. (2018). The RESTORE Randomized Controlled Trial: Impact of a Multidisciplinary Rehabilitative Program on Cardiorespiratory Fitness in Esophagogastric cancer Survivorship. *Annals of surgery, 268*(5), 747-755. <https://doi.org/https://dx.doi.org/10.1097/SLA.0000000000002895>

Ohira, T., Schmitz, K. H., Ahmed, R. L., & Yee, D. (2006). Effects of Weight Training on Quality of Life in Recent Breast Cancer Survivors: The Weight Training for Breast Cancer Survivors (WTBS) Study. *Cancer, 106*(9), 2076-2083. <https://doi.org/http://dx.doi.org/10.1002/cncr.21829http://dx.doi.org/10.1002/cncr.21829>

Park, J.-H., Lee, J., Oh, M., Park, H., Chae, J., Kim, D.-I., Lee, M. K., Yoon, Y. J., Lee, C. W., Park, S., Jones, L. W., Kim, N. K., Kim, S. I., & Jeon, J. Y. (2015). The effect of oncologists' exercise recommendations on the level of exercise and quality of life in survivors of breast and colorectal cancer: A randomized controlled trial. *Cancer, 121*(16), 2740-2748. <https://doi.org/https://dx.doi.org/10.1002/cncr.29400>

Park, S.-H., Tish Knobf, M., & Jeon, S. (2019). Endocrine Therapy-Related Symptoms and Quality of Life in Female Cancer Survivors in the Yale Fitness Intervention Trial. *Journal of nursing scholarship : an official publication of Sigma Theta Tau International Honor Society of Nursing, 51*(3), 317-325. <https://doi.org/https://dx.doi.org/10.1111/jnu.12471>

Pisu, M., Demark-Wahnefried, W., Kenzik, K. M., Oster, R. A., Lin, C. P., Manne, S., Alvarez, R., & Martin, M. Y. (2017). A dance intervention for cancer survivors and their partners (RHYTHM). *Journal of cancer survivorship : research and practice, 11*(3), 350-359. <https://doi.org/https://dx.doi.org/10.1007/s11764-016-0593-9>

Prinsen, H., Bleijenberg, G., Heijmen, L., Zwarts, M. J., Leer, J. W. H., Heerschap, A., Hopman, M. T. E., & Van Laarhoven, H. W. M. (2013). The role of physical activity and physical fitness in postcancer fatigue: A randomized controlled trial. *Supportive Care in Cancer, 21*(8), 2279-2288. <https://doi.org/10.1007/s00520-013-1784-9>

Reeves, M., Winkler, E., McCarthy, N., Lawler, S., Terranova, C., Hayes, S., Janda, M., Demark-Wahnefried, W., & Eakin, E. (2017). The Living Well after Breast Cancer TM Pilot Trial: a weight loss intervention for women following treatment for breast cancer. *Asia-Pacific journal of clinical oncology, 13*(3), 125-136. <https://doi.org/https://dx.doi.org/10.1111/ajco.12629>

Reeves, M. M., Terranova, C. O., Winkler, E. A. H., McCarthy, N., Hickman, I. J., Ware, R. S., Lawler, S. P., Eakin, E. G., & Demark-Wahnefried, W. (2021). Effect of a Remotely Delivered Weight Loss Intervention in Early-Stage Breast Cancer: Randomized Controlled Trial. *Nutrients, 13*(11). <https://doi.org/https://dx.doi.org/10.3390/nu13114091>

Rogers, L. Q., Courneya, K. S., Anton, P. M., Hopkins-Price, P., Verhulst, S., Vicari, S. K., Robbs, R. S., Mocharnuk, R., & McAuley, E. (2015). Effects of the BEAT Cancer physical activity behavior change intervention on physical activity, aerobic fitness, and quality of life in breast cancer survivors: a multicenter randomized controlled trial. *Breast cancer research and treatment, 149*(1), 109-119. <https://doi.org/https://dx.doi.org/10.1007/s10549-014-3216-z>

Rogers, L. Q., Hopkins-Price, P., Vicari, S., Markwell, S., Pamenter, R., Courneya, K. S., Hoelzer, K., Naritoku, C., Edson, B., Jones, L., Dunnington, G., & Verhulst, S. (2009). Physical activity and health outcomes three months after completing a physical activity behavior change intervention: persistent and delayed effects. *Cancer epidemiology, biomarkers & prevention : a publication of the American Association for Cancer Research, cosponsored by the American Society of Preventive Oncology, 18*(5), 1410-1418. <https://doi.org/https://dx.doi.org/10.1158/1055-9965.EPI-08-1045>

Ruiz-Vozmediano, J., Lohnchen, S., Jurado, L., Recio, R., Rodriguez-Carrillo, A., Lopez, M., Mustieles, V., Exposito, M., Arroyo-Morales, M., & Fernandez, M. F. (2020). Influence of a Multidisciplinary Program of Diet, Exercise, and Mindfulness on the Quality of Life of Stage IIA-IIB Breast Cancer Survivors. *Integrative cancer therapies, 19*(101128834), 1534735420924757. <https://doi.org/https://dx.doi.org/10.1177/1534735420924757>

Saarto, T., Penttinen, H. M., Sievanen, H., Kellokumpu-Lehtinen, P.-L., Hakamies-Blomqvist, L., Nikander, R., Huovinen, R., Luoto, R., Kautiainen, H., Jarvenpaa, S., Idman, I., Utriainen, M., Vehmanen, L., Jaaskelainen, A.-S., Elme, A., Ruohola, J., Palva, T., Vertio, H., Rautalahti, M., Fogelholm, M., Blomqvist, C., & Luoma, M.-L. (2012). Effectiveness of a 12-month exercise program on physical performance and quality of life of breast cancer survivors. *Anticancer research, 32*(9), 3875-3884. <http://ar.iiarjournals.org/content/32/9/3875.full.pdf>

<https://ar.iiarjournals.org/content/anticanres/32/9/3875.full.pdf>

Sandel, S. L., Judge, J. O., Landry, N., Faria, L., Ouellette, R., & Majczak, M. (2005). Dance and movement program improves quality-of-life measures in breast cancer survivors. *Cancer Nursing, 28*(4), 301-309. <http://ezproxy.flinders.edu.au/login?url=http://search.ebscohost.com/login.aspx?direct=true&db=cin20&AN=106528322&site=ehost-live>

Scott, E., Daley, A. J., Doll, H., Woodroofe, N., Coleman, R. E., Mutrie, N., Crank, H., Powers, H. J., & Saxton, J. M. (2013). Effects of an exercise and hypocaloric healthy eating program on biomarkers associated with long-term prognosis after early-stage breast cancer: a randomized controlled trial. *Cancer causes & control : CCC, 24*(1), 181-191. <https://doi.org/https://dx.doi.org/10.1007/s10552-012-0104-x>

Shobeiri, F., Masoumi, S. Z., Nikravesh, A., Heidari Moghadam, R., & Karami, M. (2016). The Impact of Aerobic Exercise on Quality of Life in Women with Breast Cancer: A Randomized Controlled Trial. *Journal of research in health sciences, 16*(3), 127-132. <https://www.ncbi.nlm.nih.gov/pmc/articles/PMC7191023/pdf/jrhs-16-127.pdf>

Short, C. E., James, E. L., Girgis, A., D'Souza, M. I., & Plotnikoff, R. C. (2015). Main outcomes of the Move More for Life Trial: a randomised controlled trial examining the effects of tailored-print and targeted-print materials for promoting physical activity among post-treatment breast cancer survivors. *Psycho-Oncology, 24*(7), 771-778. <https://doi.org/https://dx.doi.org/10.1002/pon.3639>

Singleton, A. C., Raeside, R., Partridge, S. R., Hyun, K. K., Tat-Ko, J., Sum, S. C. M., Hayes, M., Chow, C. K., Thiagalingam, A., Maka, K., Sherman, K. A., Elder, E., & Redfern, J. (2022). Supporting women’s health outcomes after breast cancer treatment comparing a text message intervention to usual care: the EMPOWER-SMS randomised clinical trial. *Journal of Cancer Survivorship*. <https://doi.org/10.1007/s11764-022-01209-9>

Speck, R. M., Gross, C. R., Hormes, J. M., Ahmed, R. L., Lytle, L. A., Hwang, W.-T., & Schmitz, K. H. (2010). Changes in the Body Image and Relationship Scale following a one-year strength training trial for breast cancer survivors with or at risk for lymphedema. *Breast cancer research and treatment, 121*(2), 421-430. <https://doi.org/https://dx.doi.org/10.1007/s10549-009-0550-7>

Strunk, M. A., Zopf, E. M., Steck, J., Hamacher, S., Hallek, M., & Baumann, F. T. (2018). Effects of Kyusho Jitsu on Physical Activity-levels and Quality of Life in Breast Cancer Patients. *In vivo (Athens, Greece), 32*(4), 819-824. <https://doi.org/https://dx.doi.org/10.21873/invivo.11313>

Swisher, A. K., Abraham, J., Bonner, D., Gilleland, D., Hobbs, G., Kurian, S., Yanosik, M. A., & Vona-Davis, L. (2015). Exercise and dietary advice intervention for survivors of triple-negative breast cancer: effects on body fat, physical function, quality of life, and adipokine profile. *Supportive care in cancer : official journal of the Multinational Association of Supportive Care in Cancer, 23*(10), 2995-3003. <https://doi.org/https://dx.doi.org/10.1007/s00520-015-2667-z>

Thorsen, L., Skovlund, E., Strømme, S. B., Hornslien, K., Dahl, A. A., & Fosså, S. D. (2005). Effectiveness of physical activity on cardiorespiratory fitness and health-related quality of life in young and middle-aged cancer patients shortly after chemotherapy. *Journal of Clinical Oncology, 23*(10), 2378-2388.

Toohey, K., Pumpa, K., McKune, A., Cooke, J., DuBose, K. D., Yip, D., Craft, P., & Semple, S. (2018). Does low volume high-intensity interval training elicit superior benefits to continuous low to moderate-intensity training in cancer survivors? *World Journal of Clinical Oncology, 9*(1), 1-12. <https://doi.org/10.5306/wjco.v9.i1.1>

Vallance, J. K., Courneya, K. S., Plotnikoff, R. C., Dinu, I., & Mackey, J. R. (2008). Maintenance of physical activity in breast cancer survivors after a randomized trial. *Medicine & Science in Sports & Exercise, 40*(1), 173-180. <https://doi.org/10.1249/mss.0b013e3181586b41>

Vallance, J. K., Nguyen, N. H., Moore, M. M., Reeves, M. M., Rosenberg, D. E., Boyle, T., Milton, S., Friedenreich, C. M., English, D. R., & Lynch, B. M. (2020). Effects of the ACTIVity And TEchnology (ACTIVATE) intervention on health-related quality of life and fatigue outcomes in breast cancer survivors. *Psycho-Oncology, 29*(1), 204-211. <https://doi.org/http://dx.doi.org/10.1002/pon.5298http://dx.doi.org/10.1002/pon.5298>

Vallerand, J. R., Rhodes, R. E., Walker, G. J., & Courneya, K. S. (2018). Feasibility and preliminary efficacy of an exercise telephone counseling intervention for hematologic cancer survivors: a phase II randomized controlled trial. *Journal of cancer survivorship : research and practice, 12*(3), 357-370. <https://doi.org/https://dx.doi.org/10.1007/s11764-018-0675-y>

van de Wiel, H. J., Stuiver, M. M., May, A. M., van Grinsven, S., Aaronson, N. K., Oldenburg, H. S. A., van der Poel, H. G., Koole, S. N., Retèl, V. P., van Harten, W. H., & Groen, W. G. (2021). Effects of and lessons learned from an internet-based physical activity support program (With and without physiotherapist telephone counselling) on physical activity levels of breast and prostate cancer survivors: The pablo randomized controlled trial. *Cancers, 13*(15). <https://doi.org/10.3390/cancers13153665>

von Gruenigen, V. E., Gibbons, H. E., Kavanagh, M. B., Janata, J. W., Lerner, E., Courneya, K. S., von Gruenigen, V. E., Gibbons, H. E., Kavanagh, M. B., Janata, J. W., Lerner, E., & Courneya, K. S. (2009). A randomized trial of a lifestyle intervention in obese endometrial cancer survivors: quality of life outcomes and mediators of behavior change. *Health & Quality of Life Outcomes, 7*, 17-17. <https://doi.org/10.1186/1477-7525-7-17>

Wang, L. F., Eaglehouse, Y. L., Poppenberg, J. T., Brufsky, J. W., Geramita, E. M., Zhai, S., Davis, K. K., Gibbs, B. B., Metz, J., & van Londen, G. J. (2021). Effects of a personal trainer-led exercise intervention on physical activity, physical function, and quality of life of breast cancer survivors. *Breast cancer (Tokyo, Japan), 28*(3), 737-745. <https://doi.org/https://dx.doi.org/10.1007/s12282-020-01211-y>

Willems, R. A., Bolman, C. A. W., Mesters, I., Kanera, I. M., Beaulen, A. A. J. M., & Lechner, L. (2017). Short-term effectiveness of a web-based tailored intervention for cancer survivors on quality of life, anxiety, depression, and fatigue: randomized controlled trial. *Psycho-Oncology, 26*(2), 222-230. <https://doi.org/https://dx.doi.org/10.1002/pon.4113>

Winkels, R. M., Sturgeon, K. M., Kallan, M. J., Dean, L. T., Zhang, Z., Evangelisti, M., Brown, J. C., Sarwer, D. B., Troxel, A. B., Denlinger, C., Laudermilk, M., Fornash, A., DeMichele, A., Chodosh, L. A., & Schmitz, K. H. (2017). The women in steady exercise research (WISER) survivor trial: The innovative transdisciplinary design of a randomized controlled trial of exercise and weight-loss interventions among breast cancer survivors with lymphedema. *Contemporary clinical trials, 61*(101242342), 63-72. <https://doi.org/https://dx.doi.org/10.1016/j.cct.2017.07.017>

Winters-Stone, K. M., Lyons, K. S., Dobek, J., Dieckmann, N. F., Bennett, J. A., Nail, L., & Beer, T. M. (2016). Benefits of partnered strength training for prostate cancer survivors and spouses: results from a randomized controlled trial of the Exercising Together project. *Journal of cancer survivorship : research and practice, 10*(4), 633-644. <https://doi.org/https://dx.doi.org/10.1007/s11764-015-0509-0>
