# Supplementary material for: A meta-analysis of healthy lifestyle interventions addressing quality of life of cancer survivors in the post treatment phase
Source: J Cancer Surviv. 2024 Jan 11;19(3):940–56. doi: 10.1007/s11764-023-01514-x (PMC12081566; doi:10.1007/s11764-023-01514-x)
Supplement: Supplementary file 3 — (DOCX 350 kb) [file 11764_2023_1514_MOESM3_ESM.docx]

**Table 3**

Risk of bias assessment of studies included in the meta-analysis

| Study | Randomization process | Deviations from intended interventions | Missing outcome data | Selection of the reported result | Overall |
| --- | --- | --- | --- | --- | --- |
| Broderick 2013 |  |  |  |  |  |
| Daley 2007 |  |  |  |  |  |
| Ohira 2006 |  |  |  |  |  |
| Murtezani 2014 |  |  |  |  |  |
| MCNEIL 2019 |  |  |  |  |  |
| Toohey 2018 |  |  |  |  |  |
| Fillion 2008 |  |  |  |  |  |
| Lahart 2016 |  |  |  |  |  |
| Park 2015 |  |  |  |  |  |
| Willems 2017 |  |  |  |  |  |
| Von Gruenigen 2009 |  |  |  |  |  |
| Winkels 2017 |  |  |  |  |  |
| Kampshoff 2015 |  |  |  |  |  |
| Kim 2019 |  |  |  |  |  |
| Shobeiri 2016 |  |  |  |  |  |
| Brown 2022 |  |  |  |  |  |
| Wang 2021 |  |  |  |  |  |
| Gorzelitz 2022 |  |  |  |  |  |
| Singleton 2022 |  |  |  |  |  |
| Reeves 2021 |  |  |  |  |  |
| LongParma 2022 |  |  |  |  |  |
| Demark-Wahnefried 2018 |  |  |  |  |  |
| MuleroPortela 2008 |  |  |  |  |  |
| McKenzie 2003 |  |  |  |  |  |
| Scott 2013 |  |  |  |  |  |
| Hagstrom 2016 |  |  |  |  |  |
| Casla 2015 |  |  |  |  |  |
| VandeWiel 2021 |  |  |  |  |  |
| Rogers 2015 |  |  |  |  |  |
| Galiano-Castillo 2016 |  |  |  |  |  |
| Bourke 2011 |  |  |  |  |  |
| Braakhuis 2017 |  |  |  |  |  |
| Koutoukidis 2019 |  |  |  |  |  |
| Winters-Stone 2016 |  |  |  |  |  |
| Ghavami 2017 |  |  |  |  |  |
| Vallance 2020 |  |  |  |  |  |
| Kim 2011 |  |  |  |  |  |
| Strunk 2018 |  |  |  |  |  |
| Koutoukidis 2020 |  |  |  |  |  |
| Ruiz-Vozmediano 2020 |  |  |  |  |  |
| Short 2015 |  |  |  |  |  |
| Rogers 2009 |  |  |  |  |  |
| Livingston 2015 |  |  |  |  |  |
| Golsteijn 2018 |  |  |  |  |  |
| Holtdirk 2021 |  |  |  |  |  |
| Garcia-Soidan 2020 |  |  |  |  |  |
| Ho 2020 |  |  |  |  |  |
| Kristensen 2020 |  |  |  |  |  |
| Moraes 2021 |  |  |  |  |  |
| Pisu 2017 |  |  |  |  |  |
| Blair 2021 |  |  |  |  |  |
| Mardani 2021 |  |  |  |  |  |
| McGowan 2013 |  |  |  |  |  |
| Reeves 2017 |  |  |  |  |  |
| Burnham 2002 |  |  |  |  |  |
| Littman 2012 |  |  |  |  |  |
| Bail 2018 |  |  |  |  |  |
| Basen-Engquist 2006 |  |  |  |  |  |
| Swisher 2015 |  |  |  |  |  |
| Cramer 2015 |  |  |  |  |  |
| Naumann 2012 |  |  |  |  |  |
| Culos-Reed 2010 |  |  |  |  |  |
| Culos-Reed 2006 |  |  |  |  |  |
| DeLuca 2016 |  |  |  |  |  |
| O'Neill 2018 |  |  |  |  |  |
| Dieli-Conwright 2018 |  |  |  |  |  |
